# Investigating the Relationship of Genotype and Climate Conditions on the Volatile Composition and Sensory Profile of Celery (*Apium graveolens*)

**DOI:** 10.3390/foods10061335

**Published:** 2021-06-10

**Authors:** Lucy Turner, Stella Lignou, Frances Gawthrop, Carol Wagstaff

**Affiliations:** 1 Department of Food and Nutritional Sciences, Harry Nursten Building, University of Reading, Whiteknights, Reading RG6 6DZ, UK; L.Turner@pgr.reading.ac.uk (L.T.); c.wagstaff@reading.ac.uk (C.W.); 2 A.L.Tozer Ltd., Pyports, Downside Bridge Road, Cobham KT11 3EH, UK; frances.gawthrop@tozerseeds.com

**Keywords:** celery, aroma, volatile compounds, SPME GCMS, phthalides, terpenes, preharvest

## Abstract

*Apium graveolens* is a biennial crop grown across the globe for its stalks, leaves and seed and is known for its distinct flavour and strong taste. Various extraction methods on fresh and dried celery and its essential oil are reported in the literature examining the aroma profile of this crop and demonstrating that its volatile composition is determined by variables including cultivar, season, geographical location and agronomic practices. This study investigated the volatile and sensory profile of eight celery genotypes grown over two years (2018 and 2020) in the same location in the UK. Solid-phase-micro-extraction followed by gas chromatography-mass spectrometry were used to determine the volatile compounds present in these genotypes and sensory evaluation using a trained panel to assess the sensory profile of fresh celery. Significant differences (*p* < 0.05) in the volatile composition and sensory profile were observed and influenced by both genotype and harvest year. Two genotypes exhibited similar aroma composition and sensory profile between the years. Celery samples harvested in 2018, which possessed air temperatures that were considerably warmer than in 2020, exhibited higher proportions of sesquiterpenes and phthalides and we hypothesise that the higher proportions were generated as a response to heat stress. Studying the relationship between the genotype and the environment will provide clear information to guide growers in how to consistently produce a higher quality crop.

## 1. Introduction

Celery is a vegetable that belongs to the Apiaceae family which is grown across the globe and consumed regularly and forms part of the “holy trinity” or “Soffritto” in cooking, used raw in salads or with condiments [1]. The investigation of the aroma and flavour of celery has been studied using a range of extraction techniques, such as solvent assisted flavour extraction (SAFE) and solid phase microextraction (SPME), combined with instrumental analysis, such as gas chromatography/mass spectrometry (GC/MS) on celery leaf, petiole and seed. The consensus is that terpenes (monoterpenes and sesquiterpenes) and phthalides make up the majority of compounds present in the flavour profile. Phthalides, in particular, have been shown to be key contributors to typical celery aroma (3-n-butylphthalide, sedanenolide and (E)- ligustilide and (Z)-ligustilide) and possess odour descriptors such as “celery”, “herbal” and “green” [2,3]. The composition of alcohol, aldehyde and ester compounds have been poorly represented in literature. Although they are not characteristic compounds to celery odour, their importance should not be neglected as these compounds contribute to green, fresh and woody notes that are important to the overall celery aroma. Wilson [4] identified and quantified 13 alcohols in celery essential oil using gas chromatography including n-hexanol, cis-3-hexene-1-ol and dihydrocarveol. Wilson commented on the pleasant aroma of these compounds and concluded that although they are not characteristic compounds of celery, they complete the typical flavour and aroma of celery [4].

In a recent review by the authors [5], the complexity of the aroma profile is discussed and the variation within reported datasets caused by differences in cultivar, geographical location of growth, agricultural techniques as well as extraction and analysis techniques are highlighted. In order to overcome these variances, Turner et al. [5] recommended the use of Minimum Standards About a Plant Aroma Experiment (MIAPAE), ultimately leading to a repository of data whereby accurate interpretation of results and correct experimental repetition can occur. Importantly, it was demonstrated that the genotype alone does not determine the final flavour outcome, but other factors during preharvest (cultivar, climate and agronomy) and postharvest (harvest techniques and storage conditions) simultaneously influences the final composition [5,6]. The application of alternative agronomic practices, including varying nitrogen levels in soil, the use of irrigation systems and inorganic/organic fertilisers, as well as growing celery in different geographical regions have all been shown to influence the aroma composition of celery [7,8,9,10,11]. Rożek, Nurzyńska-Wierda and Kosior [12] explained the consequences of agricultural techniques on the volatile composition of leaf celery essentials, while van Wassenhove, Dirinck, Schamp and Vulsteke [13] concluded that the use of fertiliser (organic and/or inorganic) resulted in a decrease in terpene and phthalide content.

Limited research has been conducted on the impact of the environment on the volatile composition of celery, with few studies using the same cultivar over multiple sites and seasons that are compliant to MIAPAE [5]. van Wassenhove, Dirinck, Vulsteke and Schamp [14] investigated the volatile composition of four celery cultivars grown in two seasons (1986 and 1987) on sandy loam fields in Belgium. Although differences in the composition were observed, their focus was not on the variation of composition but more on the validity of their method to identify and separate terpenes and phthalides in celery. Genotypic and seasonal differences were observed in the total terpene and phthalide content of all four cultivars [14]. Lund, Wagner and Bryan [15] also reported differences in the oil composition of celery (Utah 5270) waste trimmings between November 1972 and July 1973, yet no seasonal significant differences were shown. Conversely to van Wassenhove et al. [14], a much smaller group of compounds were investigated by Lund et al. [15] that numbered around 12 compared to the 33 compounds identified by van Wassenhove et al. [14]. This suggests that the harvest year has minimal impact over the volatile composition. Alternatively, Shojaei, Ebrahimi and Salini [10] showed the impact of the environment on the volatile composition by testing one species of wild celery (*Kelussia odoratissima*) sampled across three different regions of Iran. They identified trans-ligustilide as the main compound from the three locations contributing various percentages—47.31%, 37.55% and 33.73%. There were also variations in the presence of compounds throughout three ecotypes; the Bazoft ecotype was found to contain fewer compounds than the ecotypes grown in Koohrang and Samsani [10].

The aim of this study was to investigate the relationship between genotype and the environment on the volatile composition of eight celery genotypes grown in the UK across two different years (2018 and 2020). In addition, sensory evaluation using a trained panel was used in order to understand how chemical and physiological changes lead to differences in organoleptic perception and used to identify interactions between compounds groups and climate. Ultimately, this information could assist breeders and growers to develop and select cultivars that are optimal for specific growing climates and to allow for the production of a consistent quality product.

## 2. Materials and Methods

### 2.1. Celery Material and MIAPAE Standards

#### 2.1.1. Sample Information

The eight genotypes used in this study were chosen based on their differences in physical and chemical attributes. Although commercial confidentiality precludes revealing the exact genetic identity of each genotype used in this study, the origins of these parental breeding lines and their images postharvest can be found in the Supplementary Materials Table S1.

#### 2.1.2. Timing, Location and Environment

The celery seeds (*Apium graveolens*) of eight parental genotypes supplied by Tozer Seeds Ltd. (Cobham, UK) were grown in commercial conditions and harvested in Cambridgeshire (UK) by G’s Fresh Ltd. (Ely, UK, 52°21′12.9″ N 0°17′15.6″ E) during the spring/summer of 2018 and 2020. The celery was grown in a field with commercial celery products and treated by the same agronomic techniques and conditions as commercial celery, including identical fertiliser application and exposure to water. For both years, 20–25 mm of overhead irrigation was used and standard commercial fertiliser, pest and disease control regimes were applied. In 2018, plugs were transplanted mid-June after growing in the nursery for 22 days and then harvested 91 days later. The average daily air temperature was 18.2 °C with an average soil temperature of 23.8 °C, 0.2 mm of rainfall daily and an average relative humidity of 88.1%. In 2020, the plugs were transplanted late April after growing in the nursery for 24 days and were harvested 76 days later. The average daily air temperature was 14.3 °C with an average soil temperature of 15.4 °C, 0.05 mm daily rainfall and an average relative humidity of 74.8%. Prior to the harvest, the celery is tested regularly in-field to ensure standards for commercial quality are met, including visual and taste tests. The celeries were harvested within a close time-frame compared to the commercial produce also being grown in the field.

#### 2.1.3. Raw Material Collection, Processing and Storage

The celery was grown in three randomised blocks in the centre of the field to reduce any influence from edge effects at a density of 10 plants m^−2^ and three replicates were harvested from each block using a celery knife. Celery petioles were cut to 20 cm, discarding outer petioles, the base, leaves and any knuckles and then sealed in labelled bags for transportation to the University of Reading (United Kingdom). Celery samples used for sensory evaluation were refrigerated for one day, while samples for aroma analysis were immediately frozen at −80 °C for one week and subsequently freeze-dried for five days. Samples were then milled into a fine powder using a milling machine (Thomas Scientific, Swedesboro, NJ, USA) and then stored in an airtight container for a maximum of two weeks before analysis with gas chromatography-mass spectrometry (GC/MS).

### 2.2. Chemical Reagents

For GC/MS analysis, calcium chloride and the alkane standard C_6_–C_25_ (100 μg/mL) in diethyl ether were obtained from Merck (Poole, UK).

### 2.3. Solid Phase Microextraction (SPME) Followed by GC/MS

Celery (0.5 g) was combined with 0.5 mL of saturated calcium chloride solution and filled to 5 mL using HPLC-grade water in a 15 mL SPME vial fitted with a screw cap. Analysis was carried out by automated headspace SPME using an Agilent 110 PAL injection system and Agilent 7890 gas chromatograph with 5975C mass spectrometer (Agilent, Santa Clara, CA, USA). The SPME fibre stationary phase was composed of 75 µm divinylbenzene/Carboxen™ on polydimethylsiloxane, Supelco (Bellefonte, PA, USA). Equilibration was set for 10 min at 37 °C before exposing the fibre to the sample headspace for 30 min. Throughout equilibration and fibre exposure, the sample was constantly agitated at a rate of 500 rpm and kept at 37 °C. After extraction, the SPME device was inserted into the GC injection port and desorbed for 5 min. An Agilent capillary column HP-5MS (30 m × 250 μm × 0.25 μm thickness) (Agilent, Santa Clara, CA, USA) was used for chromatographic separation. The temperature program used was: 2 min at 80 °C isothermal, an increase of 4 °C/min to 250 °C and 6 min at 250 °C isothermal. Helium was used as the carrier gas at a flow rate of 1.2 mL/min. The temperature of the injector, interface and detector was 250 °C and the sample injection mode was splitless. Mass spectra were measured in electron ionization mode with an ionization energy of 70 eV, the scan range from 29 to 250 m/z and the scan rate of 5.3 scans/s. The data were recorded using HP G1034C Chemstation system.

Volatiles were identified by comparing each mass spectrum with spectra from authentic compounds analysed in our laboratory (The Flavour Centre, University of Reading) or from the NIST mass spectral database (NIST/EPA/NIH Mass Spectral database, 2011). To confirm the identification, the linear retention index (LRI) was calculated for each volatile compound using the retention times of a homologous series of C6–C25 n-alkanes and by comparing the LRI with those of authentic compounds analysed under similar conditions as described by Turner et al. [16].

### 2.4. Sensory Evaluation of Fresh Celery Samples

Sensory evaluation was carried out using quantitative descriptive analysis (QDA^TM^) to determine the sensory characteristics of the eight celery samples and the characteristics were estimated quantitatively. The trained sensory panel at the Sensory Science Centre (University of Reading, *n* = 12; 11 female and 1 male) was used to develop a consensus vocabulary to describe the sensory characteristics of the eight celery genotypes. During the development of the sensory profile, the panelists were asked to describe the appearance, odour, taste, flavour, mouthfeel and aftereffects of the samples in order to produce as many descriptive terms as seemed appropriate. References were used to help confirm the characteristics of certain attributes including fresh and dried fennel, salad rocket, flat leaf parsley and fresh coriander. The terms were discussed by the panelists as a group, with the help of the panel leader, and this led to a consensus of 22 and 24 attributes for the 2018 and 2020 harvest, respectively. The sensory assessment of the samples was carried out in a temperature-controlled room (22 °C) under artificial daylight and in isolated booths, each equipped with an iPad. Celery petioles were chosen to be as uniform as possible. The first outer petioles were removed and discarded. The next ring of petioles were used and these were washed with filtered water and cut to 15 cm petiole length prior to serving to the panellists at room temperature. The panellists scored in duplicate for each sample in separate sessions. Compusense Cloud Software (Version 21.0.7713.26683, Compusense, Guelph, ON, Canada) was used to acquire the data. Samples, coded with three-digit random numbers, were provided in a monadic balanced order, with sample sets randomly allocated to panelists. The panellists were asked to assess the appearance first; to break the petiole in half to assess the odour; to bite from the middle for taste, flavour and mouthfeel; and then after 30 s delay to assess the aftereffects. The intensity of each attribute for each sample was recorded on a 100 point unstructured line scale. Between samples, the panellists cleansed their palate with water and crackers.

For the 2020 harvest, due to the COVID-19 pandemic restrictions, the trained panel assessed the samples from home in July 2020. Vocabulary refreshment and training sessions occurred prior to scoring virtually on the Teams platform. Samples were prepared similarly to 2018 but were sent out to panellists using chilled transport couriers. The panellists completed their scoring simultaneously using Compusense Cloud software whilst on video on Teams.

### 2.5. Statistical Analysis

The percentage composition was calculated from the data collected by SPME GCMS analysis. Quantitative data for each compound identified in the SPME GC/MS analysis were analysed by both one-way and two-way analysis of variance (ANOVA) and principal component analysis (PCA) using XLSTAT Version 2020.1.3 (Addinsoft, Paris, France). For those compounds exhibiting significant difference in the one-way ANOVA, Tukey’s Honest Significant Difference post hoc test was applied to determine the sample means that differed significantly (*p* < 0.05) between harvest maturities and the celery genotypes. These data are shown in Table 1. Only those compounds exhibiting significant differences between harvest year, genotype and their interaction (harvest year × genotype) were included in the principal component analysis.

SENPAQ version 6.3 (Qi Statistics, Kent, UK) was used to carry out ANOVA of sensory panel data. The means from sensory data were taken over assessors and correlated with the percentage composition means from the instrumental data via PCA using XLSTAT.

## 3. Results and Discussion

### 3.1. Volatile Composition

In total, 86 compounds were identified in the headspace of the eight celery genotypes in both harvest years (2018 and 2020) and listed in Table 1. Sixty-five compounds were identified in 2018 across eight genotypes, including: 22 monoterpenes, ten sesquiterpenes, eight aldehydes, five alcohols (three of which are classified as monoterpenoid alcohols) and five phthalides. Nine additional compounds were identified in the headspace of the same genotypes from the 2020 harvest including: 22 monoterpenes, 13 sesquiterpenes, five phthalides and five alcohols (including three monoterpenoid alcohols).

Quantitative differences were observed between the two harvest years (E) as well as the eight genotypes (G) used in this study. Two-way ANOVA revealed more significant differences between aroma composition caused by the harvest year compared to the genotype, although differences caused by the genotype were still observed. The majority of alkanes and compounds including nonanal, α-thujene, camphene, sabinene, (+)-cis-p-mentha-2,8-dien-1-ol, α-ylangene, (E)-β-caryophyllene and trans-neocnidilide expressed no significant difference in the relative amount between 2018 harvest and 2020 harvest.

**Table 1:** Percentage composition of volatile compounds identified in the headspace of eight celery genotypes using SPME GC/MS and harvested in 2018 and 2020.

Code	Compound	LRI_expt_^a^	ID ^b^	Percentage Composition (%) ^c^																	*p* ^d^	
				2018								2020										
				5	8	10	12	15	18	22	25	5	8	10	12	15	18	22	25	E ^e^	G ^f^	GxE ^g^
	Alcohols																					
A1	3-methyl-3-buten-1-ol	730	A	0.42±	0.31±	0.94±	0.35±	0.22±	0.23±	0.30±	0.39±	nd ^a^	nd ^a^	nd ^a^	nd ^a^	nd ^a^	nd ^a^	nd ^a^	nd ^a^	***	***	***
				0.08 ^b^	0.04 ^ab^	0.27 ^c^	0.14 ^ab^	0.07 ^ab^	0.06 ^ab^	0.12 ^ab^	0.06 ^b^											
A2	(E)-2-penten-1-ol	758	A	0.73±	0.42±	0.64±	0.23±	0.32±	0.65±	1.2±	0.50±	tr±	tr±	0.12±	tr±	0.15±	tr±	tr±	tr±	***	***	***
				0.28 ^ab^	0.16 ^ab^	0.04 ^ab^	0.08 ^a^	0.09 ^a^	0.23 ^ab^	0.54 ^b^	0.22 ^ab^	0.01 ^a^	0.01 ^a^	0.05 ^a^	0.01 ^a^	0.03 ^a^	0.05 ^a^	0.03 ^a^	0.01 ^a^			
A3	1-pentanol	763	A	0.21±	0.11±	0.31±	0.13±	0.23±	0.39±	0.63±	0.28±	tr±	tr±	tr±	tr±	0.10±	0.14±	0.12±	0.10±	**	**	**
				0.06 ^ab^	0.04 ^a^	0.20 ^ab^	0.10 ^a^	0.15 ^ab^	0.14 ^ab^	0.25 ^b^	0.08 ^ab^	0.01 ^a^	0.01 ^a^	0.03 ^a^	0.01 ^a^	0.03 ^a^	0.02 ^a^	0.03 ^a^	0.02 ^a^			
	Total			1.4	0.84	1.9	0.71	0.77	1.3	2.1	1.2	0.07	0.06	0.18	0.03	0.25	0.21	0.3	0.13			
	Aldehydes																					
AL1	hexanal	800	A	9.7±	1.3±	2.6±	0.65±	2.0±	8.9±	13±	6.3±	0.16±	0.11±	0.22±	0.14±	0.24±	0.35±	0.22±	0.26±	*	ns	*
				0.8	0.46	0.32	0.29	0.39	2.7	5.5	1.2	0.05	0.02	0.1	0.03	0.03	0.25	0.05	0.15			
AL2	(E)-2-hexenal	849	A	0.18±	tr±	tr±	tr±	tr±	0.15±	0.20±	0.11 ±	nd	nd	nd	nd	nd	nd	nd	nd	**	ns	**
				0.11	0.02	0.02	0.01	0.03	0.11	0.08	0.05											
AL3	heptanal	901	A	tr±	nd	0.28±	0.16±	0.25±	0.23±	0.29±	0.25±	nd	nd	nd	nd	nd	nd	nd	nd	**	ns	**
				0.03		0.15	0.13	0.16	0.14	0.08	0.15											
AL4	(E)-2-heptenal	954	A	0.10±	1.6±	1.6±	0.5±	1.5±	3.2±	4.2±	1.8±	0.18±	0.2±	0.28±	0.36±	0.54±	0.53±	0.46±	0.03±	***	***	***
				0.22 ^a^	0.55 ^abc^	0.23 ^abc^	0.04 ^ab^	0.10 ^abc^	1.5 ^bc^	1.3 ^c^	0.97 ^abc^	0.05 ^a^	0.07 ^a^	0.10 ^a^	0.04 ^ab^	0.06 ^ab^	0.16 ^bc^	0.11 ^a^	0.04 ^a^			
AL5	n-octanal	1003	A	0.10±	nd	0.49±	0.27±	0.39±	0.51±	0.51±	0.51±	0.18±	0.16±	0.22±	0.25±	0.19±	0.24±	0.25±	0.15±	*	*	*
				0.07		0.06	0.06	0.19	0.26	0.17	0.23	0.02	0.05	0.04	0.02	0.03	0.03	0.14	0.03			
AL7	m-tolualdehyde	1086	B [17]	0.33±	0.24±	4.0±	1.1±	0.95±	0.19±	0.26±	1.6±	tr±	tr±	tr±	tr±	tr±	tr±	tr±	nd ^a^	***	***	***
				0.07 ^ab^	0.02 ^a^	0.28 ^d^	0.28 ^c^	0.02 ^bc^	0.02 ^a^	0.05 ^a^	0.29 ^c^	0.01 ^a^	0.01 ^a^	0.01 ^a^	0.01 ^a^	0.01 ^a^	0.01 ^a^	0.01 ^a^				
AL8	nonanal	1105	A	0.33±	0.12±	0.20±	tr±	0.17±	0.16±	0.22±	0.19±	0.10 ±	tr±	0.21±	tr±	tr±	0.11±	0.14±	tr±	ns	ns	ns
				0.14	0.02	0.03	0.01	0.03	0.1	0.17	0.09	0.02	0.05	0.01	<0.01	0.01	0.02	0.01	0.01			
AL9	(E,E)-2,6-nonadienal	1156	A	0.21±	0.30±	0.18±	0.18±	0.17±	0.16±	tr±	0.22±	nd ^a^	nd ^a^	nd ^a^	nd ^a^	nd ^a^	nd ^a^	nd ^a^	nd ^a^	***	***	***
				0.04 ^c^	0.03 ^c^	0.02 ^bc^	0.04 ^bc^	0.03 ^bc^	0.08 ^a^	0.03 ^ab^	0.08 ^c^											
	Total			11	3.6	9.4	3	5.5	14	19	11	0.65	0.57	0.94	0.82	1.1	1.3	1.1	0.52			
	Esters																					
E1	methyl butanoate	717	A	tr ±	tr±	tr±	tr±	tr±	tr±	tr±	tr±	nd	tr±	nd	tr±	tr±	tr±	tr±	tr±	ns	ns	ns
				0.03	0.01	0.02	<0.01	0.02	0.04	0.05	0.01		<0.01		<0.01	<0.01	<0.01	<0.01	<0.01			
E2	1-octen-3-yl-acetate	1108	B [18]	nd ^a^	nd ^a^	nd ^a^	nd ^a^	nd ^a^	nd ^a^	nd ^a^	nd ^a^	tr±	tr±	0.11 ±	tr±	tr±	tr±	nd ^a^	tr±	***	***	***
												0.02 ^a^	0.01 ^a^	0.03 ^c^	0.01 ^ab^	0.01 ^a^	0.01 ^ab^		0.02 ^b^			
E3	(E)-pinocarvyl acetate	1310	B [19]	nd ^a^	nd ^a^	nd ^a^	nd ^a^	nd ^a^	nd ^a^	nd ^a^	nd ^a^	0.36±	0.38±	0.43±	0.14±	0.43±	0.55±	0.21±	0.24±	***	ns	***
												0.18 ^ab^	0.19 ^ab^	0.12 ^ab^	0.01 ^ab^	0.18 ^ab^	0.28 ^b^	0.07 ^ab^	0.05 ^ab^			
E4	carveol acetate	1343	B [20]	nd ^a^	nd ^a^	nd ^a^	nd ^a^	nd ^a^	nd ^a^	nd ^a^	nd ^a^	tr±	0.12±	0.20±	0.10 ±	0.18±	0.10 ±	tr±	0.10±	***	***	***
												0.02 ^cd^	0.05 ^bcd^	0.04 ^d^	0.01 ^ab^	0.05 ^cd^	0.02 ^bc^	0.01 ^ab^	0.02 ^abc^			
E5	hexy isobutanoate	1378	B [21]	0.10±	0.10±	0.14±	tr±	tr±	0.16±	0.32±	0.12±	nd ^a^	nd ^a^	nd ^a^	nd ^a^	nd ^a^	nd ^a^	nd ^a^	nd ^a^	***	***	***
				0.03 ^a^	0.04 ^a^	0.02 ^ab^	0.03 ^a^	0.05 ^a^	0.04 ^ab^	0.06 ^b^	0.03 ^ab^											
	Total			0.14	0.1	0.2	0.07	0.11	0.19	0.36	0.14	0.44	0.52	0.74	0.27	0.65	0.72	0.26	0.4			
	Alkanes																					
ALK1	nonane	900	A	0.41±	0.32±	0.43±	0.14±	0.13±	0.28±	nd ^a^	0.17±	0.20±	0.38±	0.71±	0.36±	0.51±	0.39±	0.29±	0.27±	*	*	*
				0.15 ^ab^	0.11 ^ab^	0.19 ^ab^	0.18 ^ab^	0.10 ^ab^	0.11 ^ab^		0.02 ^ab^	0.11 ^ab^	0.14 ^ab^	0.29 ^b^	0.11 ^ab^	0.07 ^ab^	0.22 ^ab^	0.05 ^ab^	0.04 ^ab^			
ALK2	decane	1000	A	0.80±	0.49±	nd ^a^	0.37±	0.60±	1.1±	1.7±	0.83±	0.14±	0.13±	0.10 ±	tr±	0.18±	0.31±	0.19±	0.14±	***	***	***
				0.24 ^bcd^	0.13 ^abcd^		0.11 ^abc^	0.26 ^abcd^	0.21 ^de^	0.29 ^e^	0.33 ^cd^	0.02 ^ab^	0.02 ^ab^	0.11 ^a^	0.08 ^a^	0.02 ^a^	0.01 ^abc^	0.02 ^abc^	0.01 ^ab^			
ALK3	undecane	1100	A	0.26±	0.14±	0.19±	tr±	0.24±	0.14±	tr±	0.11 ±	nd	nd	nd	nd	nd	nd	nd	nd	**	ns	ns
				0.15	0.09	0.11	0.05	0.06	0.1	0.08	0.06											
ALK4	dodecane	1199	A	0.48±	0.37±	0.46±	0.31±	0.33±	0.44±	0.46±	0.44±	0.39±	0.38±	0.18±	0.10±	0.11±	0.11±	0.10±	0.08±	ns	ns	ns
				0.08	0.03	0.05	0.1	0.1	0.13	0.1	0.12	0.36	0.34	0.11	0.08	0.1	0.04	0.09	0.04			
ALK5	tridecane	1299	A	nd	nd	nd	nd	nd	nd	nd	nd	0.61±	0.58±	0.23±	0.14±	0.13±	0.11 ±	0.10 ±	tr±	ns	ns	ns
												0.67	0.68	0.17	0.11	0.08	0.06	0.06	0.04			
ALK6	tetradecane	1399	A	0.11 ±	tr±	tr±	tr±	0.10±	0.10±	tr±	0.10 ±	0.50±	0.49±	0.28±	0.22±	tr±	0.14±	0.14±	0.11±	ns	ns	ns
				0.02	0.03	0.02	0.03	0.06	0.03	0.03	0.02	0.48	0.21	0.23	0.1	0.03	0.05	0.07	0.06			
ALK7	pentadecane	1499	A	nd	nd	nd	nd	nd	nd	nd	nd	0.25±	0.27±	0.18±	0.15±	0.17±	0.12±	0.14±	0.12±	**	ns	ns
												0.19	0.19	0.08	0.08	0.04	0..02	0.04	0.03			
ALK8	hexadecane	1600	A	nd	nd	nd	nd	nd	nd	nd	nd	0.10±	0.10 ±	0.10 ±	tr±	tr±	tr±	tr±	tr±	**	ns	ns
												0.06	0.06	0.03	0..03	0.02	0.01	0.01	0.01			
ALK9	heptadecane	1700	A	nd	nd	nd	nd	nd	nd	nd	nd	tr±	tr±	tr±	tr±	0.72±	0.69±	tr±	tr±	ns	ns	ns
												0.01	0.02	0.02	<0.01	0.12	0.39	0.01	0.01			
ALK10	octadecane			nd	nd	nd	nd	nd	nd	nd	nd	tr±	tr±	tr±	nd	nd	nd	nd	nd	ns	ns	ns
												0.01	0.01	0.01								
	Total			2.1	1.4	1.1	0.94	1.4	2.1	2.3	1.6	2.2	2.5	1.8	1.1	1.9	1.9	0.95	0.86			
	Monoterpenes																					
M1	α-thujene	933	B [22]	0.27±	0.24±	0.29±	0.30±	0.22±	0.41±	0.32±	0.22±	0.11±	0.10 ±	0.10±	0.14±	0.11±	0.24±	0.15±	0.14±	ns	ns	ns
				0.09	0.08	0.13	0.11	0.1	0.19	0.14	0.13	0.02	0.02	0.02	0.02	0.01	0.02	0.02	0.02			
M2	α-pinene	943	A	0.62±	0.85±	0.52±	0.62±	1.0±	0.89±	0.43±	0.62±	0.26±	0.14±	0.20±	tr±	0.10±	0.15±	0.12±	0.40±	***	ns	***
				0.05 ^abcd^	0.22 ^bcd^	0.19 ^abcd^	0.18 ^abcd^	0.42 ^d^	0.20 ^cd^	0.20 ^ab^	0.31 ^abcd^	0.04 ^abcd^	0.11 ^abc^	0.09 ^abc^	0.01 ^a^	0.01 ^ab^	0.01 ^abc^	0.01 ^a^	0.09 ^abcd^			
M3	camphene	960	A	2.5±	0.33±	0.29±	0.21±	0.35±	0.48±	0.66±	0.22±	0.11±	0.13±	0.17±	0.16±	0.22±	0.45±	0.28±	0.10 ±	ns	ns	ns
				0.5	0.07	0.12	0.08	0.1	0.05	0.26	0.08	0.01	0.04	0.02	0.06	0.07	0.03	0.09	0.03			
M4	sabinene	981	A	0.44±	0.33±	0.66±	0.27±	0.28±	0.45±	0.53±	0.36±	0.27±	0.25±	0.32±	0.39±	0.22±	0.49±	0.29±	0.23±	ns	ns	ns
				0.13	0.04	0.39	0.04	0.05	0.03	0.13	0.06	0.02	0.01	0.04	0.03	0.08	0.05	0.05	0.04			
M5	β-pinene	989	A	3.0±	5.2±	0.96±	5.4±	3.8±	2.7±	0.79±	4.5±	2.8±	3.9±	1.7±	5.5±	3.8±	0.13±	3.1±	4.8±	**	**	**
				0.64 ^ab^	1.6 ^b^	0.36 ^ab^	1.6 ^b^	1.6 ^ab^	0.99 ^ab^	0.24 ^ab^	1.1 ^ab^	0.8 ^ab^	1.1 ^ab^	0.39 ^ab^	0.69 ^b^	0.84 ^ab^	0.02 ^a^	0.17 ^ab^	1.1 ^ab^			
M6	myrcene	992	A	1.1±	1.9±	2.1±	2.6±	1.6±	2.1±	0.84±	1.1±	1.9±	2.6±	7.3±	7.9±	2.0±	1.9±	1.7±	2.1±	***	***	***
				0.26 ^a^	0.64 ^a^	0.74 ^a^	0.22 ^a^	0.37 ^a^	0.61 ^a^	0.34 ^a^	0.45 ^a^	0.11 ^a^	0.48 ^a^	0.65 ^b^	0.53 ^b^	0.76 ^a^	0.08 ^a^	0.27 ^a^	0.26 ^a^			
M7	α-phellandrene	1013	A	nd ^a^	nd ^a^	nd ^a^	nd ^a^	nd ^a^	nd ^a^	nd ^a^	nd ^a^	0.33±	0.31±	0.39±	0.30±	0.40±	0.53±	0.53±	0.43±	***	***	***
												0.02 ^bc^	0.03 ^b^	0.03 ^cd^	0.01 ^b^	0.03 ^cd^	0.03 ^e^	0.02 ^e^	0.03 ^d^			
M8	delta-3-carene	1019	A	0.24±	0.23±	0.25±	0.25±	0.22±	0.21±	0.32±	0.23±	tr±	tr±	tr±	tr±	nd ^a^	0.13±	nd ^a^	tr±	**	ns	**
				0.10 ^ab^	0.18 ^ab^	0.04 ^ab^	0.12 ^ab^	0.11 ^ab^	0.10 ^ab^	0.09 ^b^	0.05 ^ab^	0.01 ^ab^	0.01 ^ab^	0.01 ^a^	0.01 ^ab^		0.10 ^ab^		0.02 ^ab^			
M9	α -terpinene	1025	A	nd ^a^	nd ^a^	nd ^a^	nd ^a^	nd ^a^	nd ^a^	nd ^a^	nd ^a^	0.46±	0.42±	0.37±	0.35±	0.32±	0.37±	0.30±	0.48±	***	ns	***
												0.08 ^b^	0.11 ^b^	0.06 ^b^	0.02 ^b^	0.03 ^b^	0.15 ^b^	0.02 ^b^	0.07 ^b^			
M10	m-cymene	1032	A	4.3±	3.6±	3.5±	3.8±	3.4±	5.0±	2.8±	3.7±	8.9±	6.6±	5.4±	7.9±	4.2±	7.3±	5.8±	6.0±	***	***	***
				0.61 ^abcd^	0.41 ^abc^	0.69 ^ab^	0.43 ^abc^	0.78 ^ab^	0.71 ^abcde^	0.61 ^a^	0.55 ^abc^	1.4 ^f^	2.0 ^cdef^	0.28 ^abcde^	0.27 ^ef^	0.24 ^abcd^	0.20 ^def^	0.68 ^abcdef^	0.47 ^bcdef^			
M11	limonene	1034	A	39±	43±	33±	32±	39±	32±	29±	33±	54±	58±	59±	46±	65±	59±	61±	59±	***	***	***
				8.2 ^ab^	0.56 ^abc^	5.1 ^a^	2.3 ^a^	3.1 ^ab^	4.5 ^a^	3.9 ^cd^	3.1 ^a^	2.9 ^bcd^	4.5 ^bcd^	2.1 ^cd^	0.27 ^abc^	2.7 ^d^	2.1 ^cd^	1.6 ^cd^	1.9 ^cd^			
M12	β-(E)-ocimene	1049	B [23]	0.19±	0.18±	0.17±	0.24±	0.17±	0.16±	0.42±	0.18±	0.39±	0.25±	0.32±	0.46±	0.34±	0.28±	1.2±	0.42±	***	***	***
				0.03 ^a^	0.07 ^a^	0.05 ^a^	0.03 ^a^	0.02 ^a^	0.02 ^a^	0.08 ^a^	0.02 ^a^	0.04 ^a^	0.06 ^a^	0.11 ^a^	0.05 ^a^	0.08 ^a^	0.04 ^a^	0.22 ^b^	0.09 ^a^			
M13	γ-terpinene	1066	A	4.2±	4.3±	3.6±	5.9±	5.6±	5.5±	2.1±	5.6±	17±	16±	10±	15±	8.0±	13±	9.3±	14±	***	***	***
				1.2 ^ab^	1.2 ^ab^	0.60 ^a^	0.28 ^abcd^	0.27 ^abc^	1.4 ^abc^	0.90 ^a^	1.4 ^abc^	0.86 ^f^	1.6 ^f^	1.5 ^de^	0.67 ^f^	0.36 ^bcd^	1.3 ^ef^	0.60 ^ef^	0.27 ^f^			
M14	terpinolene	1097	A	0.62±	0.89±	0.53±	0.43±	0.36±	0.73±	0.57±	0.9±	0.75±	0.73±	0.76±	0.69±	0.79±	0.82±	0.84±	0.86±	*	ns	ns
				0.19	0.07	0.09	<0.01	0.22	0.2	0.14	0.31	0.08	0.11	0.05	0.06	0.11	0.04	0.16	0.12			
M15	allo-ocimene	1132	B [24]	0.11±	0.10 ±	0.10 ±	0.31±	0.24±	0.13±	0.31±	0.13±	0.33±	0.14±	0.23±	0.57±	0.29±	0.27±	1.7±	0.41±	***	***	***
				0.06 ^a^	0.01 ^a^	0.05 ^a^	0.03 ^ab^	0.01 ^ab^	0.04 ^ab^	0.27 ^ab^	0.08 ^ab^	0.12 ^ab^	0.07 ^ab^	0.03 ^ab^	0.03 ^b^	0.01 ^ab^	0.05 ^ab^	0.36 ^c^	0.04 ^ab^			
M16	*p*-mentha-1,5,8-triene	1135	B [22]	0.26±	0.10 ±	0.22±	0.56±	0.26±	0.13±	0.49±	0.19±	0.10±	tr±	tr±	0.12±	0.10 ±	0.10 ±	0.34±	0.10 ±	***	***	***
				0.05 ^abc^	0.01 ^ab^	0.02 ^abc^	0.09 ^d^	0.07 ^abc^	0.09 ^ab^	0.17 ^cd^	0.08 ^ab^	0.02 ^ab^	0.02 ^a^	0.01 ^ab^	0.01 ^ab^	<0.01 ^ab^	<0.01 ^ab^	0.11 ^bcd^	<0.01 ^ab^			
M17	pentylcyclohexa-1,3-diene	1166	B [19]	0.21±	0.23±	0.25±	0.46±	0.31±	0.06 ±	0.26±	0.20±	0.36±	0.34±	0.23±	0.34±	0.27±	0.18±	0.22±	0.25±	*	*	*
				0.05 ^ab^	0.08 ^ab^	0.03 ^ab^	0.11 ^b^	0.03 ^ab^	0.04 ^a^	0.16 ^ab^	0.01 ^ab^	0.09 ^b^	0.12 ^ab^	0.01 ^ab^	0.10 ^ab^	0.02 ^ab^	0.02 ^ab^	0.02 ^ab^	0.02 ^ab^			
M18	dihydrocarvone trans	1208	A	0.39±	0.36±	0.35±	0.19±	0.27±	0.18±	0.20±	0.26±	tr±	0.10±	0.10 ±	tr±	0.10 ±	tr±	0.10 ±	tr±	***	*	***
				0.09 ^e^	0.05 ^de^	0.08 ^de^	0.06 ^abcde^	0.05 ^cde^	0.04 ^abcd^	0.08 ^abcde^	0.02 ^bcde^	0.02 ^ab^	0.01 ^abc^	0.02 ^abc^	0.01 ^a^	0.03 ^abc^	0.01 ^a^	0.02 ^abc^	0.01 ^a^			
M19	carveol trans	1217	B [19]	0.23±	nd	0.10 ±	nd	0.10 ±	0.10 ±	0.16±	0.13±	0.10±	0.13±	0.19±	0.10 ±	0.15±	0.10±	0.10 ±	0.10 ±	*	ns	ns
				0.05		0.06		0.05	0.06	0.06	0.08	0.01	0.03	0.06	0.01	0.01	0.02	0.01	<0.01			
M20	(E)-dihydrocarvone	1240	B [25]	0.79±	0.79±	0.67±	0.41±	0.57±	0.43±	0.38±	0.59±	nd ^a^	nd ^a^	nd ^a^	nd ^a^	nd ^a^	nd ^a^	nd ^a^	nd ^a^	***	***	***
				0.12 ^d^	0.14 ^d^	0.10 ^cd^	0.08 ^bc^	0.09 ^bcd^	0.05 ^bc^	0.06 ^b^	0.03 ^bcd^											
M21	L-carvone	1248	A	0.43±	0.36±	0.24±	0.18±	0.23±	0.34±	0.44±	0.29±	0.22±	0.14±	0.10 ±	tr±	tr±	nd	tr±	nd	**	ns	ns
				0.19	0.1	0.02	0.03	0.08	0.15	0.07	0.06	0.03	0.04	0.01	0.02	0.01		0.03				
M22	D-carvone	1262	A	0.96±	0.57±	1.5±	0.71±	0.81±	0.61±	0.75±	1.1±	0.20±	0.12±	tr±	0.10 ±	0.10 ±	0.21±	0.15±	0.10 ±	***	***	***
				0.19 ^cd^	0.11 ^abc^	0.05 ^d^	0.06 ^abc^	0.13 ^bcd^	0.14 ^abc^	0.17 ^abc^	0.12 ^cd^	0.01 ^ab^	0.02 ^ab^	0.02 ^a^	0.01 ^abc^	0.01 ^a^	0.01 ^ab^	0.02 ^ab^	0.01 ^abc^			
M23	thymol	1290	A	0.17±	0.11±	0.12±	0.15±	0.10±	0.10±	nd ^a^	0.14±	nd ^a^	nd ^a^	nd ^a^	nd ^a^	nd ^a^	nd ^a^	nd ^a^	nd ^a^	***	***	***
				0.05 ^c^	0.14 ^bc^	0.04 ^bc^	0.09 ^c^	0.08 ^ab^	0.03 ^bc^		0.11 ^bc^											
M24	carvacrol	1317	A	0.54±	0.42±	0.45±	0.60 ±	0.29±	0.39 ±	0.18±	0.52±	nd ^a^	tr±	tr±	tr±	tr±	tr±	tr±	tr±	***	***	***
				0.08 ^e^	0.09 ^cde^	0.03 ^de^	0.02 ^e^	0.03 ^bcd^	0.03 ^cde^	0.04 ^abc^	0.04 ^de^		0.01 ^a^	0.01 ^a^	0.01 ^a^	0.01 ^a^	0.01 ^a^	0.01 ^ab^	0.01 ^a^			
	Total			61	64	50	56	59	53	42	54	89	90	87	86	87	86	87	90			
	Monoterpenoid Alcohols																					
MA1	(+)-cis-p-mentha-2,8-dien-1-ol	1122	A	0.10±	0.15±	tr±	0.28 ±	0.10±	0.10±	tr±	0.14 ±	tr±	tr±	tr±	tr±	tr±	nd	tr±	tr±	ns	ns	ns
				0.03	0.01	0.03	0.03	0.02	0.04	0.03	0.01	0.01	0.01	0.02	0.01	0.01		0.01	0.01			
MA2	dihydrolinalool	1142	A	nd ^a^	nd ^a^	nd ^a^	nd ^a^	nd ^a^	nd ^a^	nd ^a^	nd ^a^	nd ^a^	nd ^a^	tr±	tr±	nd ^a^	nd ^a^	tr±	nd ^a^	***	***	***
														0.01 ^a^	0.01 ^b^			0.01 ^a^				
MA3	trans-pinocarveol	1147	B [26]	0.59±	0.63±	0.30±	0.20±	0.28±	0.35±	tr±	0.45±	nd ^a^	nd ^a^	nd ^a^	nd ^a^	nd ^a^	nd ^a^	nd ^a^	nd ^a^	***	***	***
				0.13 ^c^	0.17 ^c^	0.08 ^abc^	0.08 ^ab^	0.02 ^abc^	0.21 ^abc^	0.03 ^a^	0.10 ^bc^											
MA4	terpinen-4-ol	1184	A	0.10±	nd ^a^	tr±	tr±	tr±	tr±	nd ^a^	0.13±	nd ^a^	nd ^a^	nd ^a^	nd ^a^	nd ^a^	nd ^a^	nd ^a^	nd ^a^	***	***	***
				0.01 ^bc^		0.03 ^ab^	0.03 ^abc^	0.03 ^ab^	0.07 ^abc^		0.03 ^c^											
MA5	(E)-8-hydroxylinalool	1349	B [19]	nd ^a^	nd ^a^	nd ^a^	nd ^a^	nd ^a^	nd ^a^	nd ^a^	nd ^a^	tr±	0.10±	0.10±	tr±	0.10±	tr±	tr±	tr±	***	***	***
												0.01 ^ab^	0.03 ^bc^	0.01 ^c^	0.01 ^ab^	0.01 ^c^	0.01 ^ab^	0.01 ^a^	0.01 ^ab^			
	Total			0.79	0.78	0.38	0.53	0.39	0.48	0.06	0.72	0.05	0.13	0.16	0.09	0.09	0.03	0.05	0.05			
	Sesquiterpenes																					
S1	α-ylangene	1384	B [22]	0.26±	0.24±	0.17±	tr±	0.16±	0.19±	0.20±	0.20±	0.10±	0.32±	0.27±	0.26±	0.16±	0.23±	0.16±	0.27±	ns	ns	ns
				0.11	0.07	0.11	0.01	0.05	0.1	0.26	0.14	0.03	0.25	0.07	0.1	0.07	0.06	0.06	0.08			
S2	α-copaene	1390	A	1.1 ±	0.86 ±	0.62 ±	0.10 ±	0.15 ±	0.49 ±	0.78 ±	0.77 ±	tr±	0.39±	0.30±	tr±	tr±	0.17±	0.30±	0.42±	***	***	***
				0.02 ^e^	0.01 ^de^	0.03 ^bcde^	0.02 ^a^	0.05 ^ab^	0.03 ^abcd^	0.04 ^cde^	0.05 ^cde^	<0.01 ^a^	0.31 ^abcd^	0.05 ^abc^	0.01 ^a^	0.01 ^ab^	0.03 ^ab^	0.10 ^abc^	0.09 ^abcd^			
S3	(E)-β-caryophyllene	1430	B [27]	tr±	tr±	nd	nd	tr±	nd	nd	nd	tr±	tr±	tr±	tr±	nd	nd	nd	nd	ns	ns	ns
				0.03	0.02			0.04				0.01	0.01	0.01	0.01							
S4	β-caryophyllene	1445	A	4.4±	5.5±	4.1±	2.5±	4.3±	4.1±	2.4±	2.2±	2.3±	2.9±	2.4±	1.3±	1.7±	2.0±	0.89±	0.97±	***	***	***
				0.61 ^cd^	0.32 ^d^	0.43 ^bcd^	0.39 ^abc^	1.3 ^cd^	1.2 ^bcd^	0.29 ^abc^	0.50 ^abc^	0.37 ^abc^	0.66 ^abc^	0.22 ^abc^	0.52 ^a^	0.29 ^ab^	0.45 ^abc^	0.06 ^a^	0.19 ^a^			
S5	(+)-aromadendrene	1452	A	0.17±	0.21±	0.15±	tr±	0.13±	0.15±	tr±	0.10±	0.10 ±	0.10 ±	0.10±	tr±	tr±	tr±	tr±	tr±	***	***	***
				0.04 ^de^	0.01 ^e^	0.04 ^cde^	0.07 ^abc^	0.03 ^abcde^	0.08 ^bcde^	0.06 ^abc^	0.01 ^abcd^	0.02 ^abc^	0.02 ^abcd^	0.02 ^abcd^	0.01 ^a^	0.01 ^a^	0.01 ^abc^	<0.01 ^a^	0.01 ^ab^			
S6	curcumene	1472	B [28]	0.18±	0.23±	0.19±	tr±	0.15±	0.22±	tr±	0.12±	tr±	0.10 ±	tr±	tr±	nd ^a^	nd ^a^	nd ^a^	nd ^a^	***	**	***
				0.09 ^cde^	0.11 ^e^	0.06 ^de^	0.05 ^abcde^	0.22 ^bcde^	0.19 ^e^	0.03 ^abcde^	0.05 ^abcde^	0.01 ^abc^	0.01 ^abcd^	0.01 ^abc^	0.01 ^ab^							
S7	α-humulene	1479	A	0.42±	0.70±	0.38±	0.49±	0.51±	0.40±	0.18±	0.26±	0.30±	0.51±	0.24±	0.30±	0.40±	0.14±	0.12±	0.14±	***	***	***
				0.16 ^abc^	0.58 ^c^	0.29 ^abc^	1.1 ^abc^	0.76 ^bc^	0.65 ^abc^	1.2 ^ab^	0.9 ^ab^	0.14 ^abc^	0.04 ^abc^	0.06 ^ab^	0.09 ^ab^	0.06 ^abc^	0.03 ^ab^	0.01 ^a^	0.01 ^ab^			
S8	α-gurjunene	1495	B [29]	nd ^a^	nd ^a^	nd ^a^	nd ^a^	nd ^a^	nd ^a^	nd ^a^	nd ^a^	0.10 ± 0.02 ^bc^	0.10 ± 0.01 ^bc^	0.10± <0.01 ^bc^	0.10 ± 0.01 ^ab^	0.10 ± 0.01 ^bc^	0.10± 0.02 ^bc^	0.10± 0.03 ^c^	0.10± 0.01 ^bc^	***	ns	***
															
S9	β-selinene	1508	B [30]	3.0±	2.7±	1.5±	4.6±	2.2±	1.9±	3.3±	3.0±	2.5±	1.6±	0.96±	1.4±	1.2±	0.85±	1.1±	1.7±	***	***	***
				0.05 ^ab^	0.06 ^ab^	0.02 ^a^	0.15 ^b^	0.19 ^ab^	0.12 ^a^	0.26 ^ab^	0.14 ^ab^	0.62 ^ab^	0.12 ^a^	0.16 ^a^	0.28 ^a^	0.32 ^a^	0.16 ^a^	0.23 ^a^	0.33 ^a^			
S10	valencene	1514	A	nd ^a^	nd ^a^	nd ^a^	2.9±	nd ^a^	nd ^a^	nd ^a^	0.20±	0.15±	0.15±	0.10±	2.6±	0.10±	0.10 ±	0.12±	0.18±	***	***	***
							0.44 ^b^				0.07 ^a^	0.21 ^a^	0.19 ^a^	0.01 ^a^	0.40 ^b^	0.05 ^a^	0.07 ^a^	0.04 ^a^	0.08 ^a^			
S11	α-selinene	1515	B [31]	0.61 ±	0.60 ±	0.43 ±	0.63±	0.54 ±	0.44±	0.71 ±	0.59±	0.28±	0.31±	0.29±	0.23±	0.22±	0.13±	0.23±	0.33±	***	ns	***
				0.02 ^bc^	0.06 ^bc^	0.05 ^abc^	0.44 ^bc^	0.04 ^abc^	0.03 ^abc^	0.02 ^c^	0.01 ^abc^	0.06 ^abc^	0.09 ^abc^	0.04 ^abc^	0.05 ^ab^	0.05 ^ab^	0.08 ^a^	0.06 ^ab^	0.03 ^abc^			
S12	kessane	1557	B [19]	nd ^a^	0.12±	nd ^a^	2.8±	nd ^a^	nd ^a^	nd ^a^	nd ^a^	0.26±	0.12±	tr±	1.7±	0.10 ±	tr±	tr±	tr±	***	***	***
					0.02 ^a^		0.05 ^c^					0.03 ^a^	0.09 ^ab^	0.01 ^a^	0.21 ^b^	0.01 ^a^	0.01 ^ab^	0.01 ^b^	0.01 ^a^			
S13	β-gurjuene ^$^	1560	B [29]	nd ^a^	nd ^a^	nd ^a^	nd ^a^	nd ^a^	nd ^a^	nd ^a^	nd ^a^	tr±	tr±	nd ^a^	tr±	tr±	tr±	nd ^a^	nd ^a^	***	***	***
												0.01 ^b^	0.01 ^ab^		0.03 ^c^	0.01 ^ab^	0.01 ^ab^					
	Total			10	11	7.5	14	8.2	7.9	7.7	7.4	6.1	6.6	4.8	8	3.9	3.8	3	4.2			
	*Phthalides*																					
P1	3-butylhexahydro phthalide	1662	B [19]	nd ^a^	nd ^a^	nd ^a^	nd ^a^	nd ^a^	nd ^a^	nd ^a^	nd ^a^	tr±	tr±	tr±	tr±	tr±	tr±	tr±	tr±	***	ns	***
												0.01 ^abc^	0.01 ^ab^	0.01 ^abc^	0.01 ^ab^	0.01 ^ab^	0.01 ^bc^	0.01 ^bc^	0.01 ^ab^			
P2	3-n-butylphthalide	1676	A	5.0±	5.2±	9.4±	6.6±	7.1±	6.7±	9.8±	7.0±	0.73±	0.52±	0.93±	0.88±	0.67±	0.93±	1.6±	1.0±	***	*	***
				0.01 ^b^	0.03 ^b^	0.05 ^c^	0.01 ^bc^	0.03 ^bc^	0.01 ^bc^	0.06 ^c^	0.03 ^bc^	0.39 ^a^	0.28 ^a^	0.30 ^a^	0.28 ^a^	0.43 ^a^	0.60 ^a^	0.40 ^a^	0.30 ^a^			
P3	(Z)-3-butylidenephthalide	1685	B [19]	0.15±	0.18±	0.36±	0.15±	0.23±	0.17±	0.25±	0.18±	nd ^a^	nd ^a^	nd ^a^	nd ^a^	nd ^a^	nd ^a^	nd ^a^	nd ^a^	***	***	***
				0.06 ^b^	0.05 ^b^	0.09 ^c^	0.02 ^bc^	0.02 ^b^	0.07 ^b^	0.34 ^bc^	0.25 ^b^											
P4	sedanenolide	1748	A	4.8±	9.7±	15±	16±	14±	9.5±	11±	13±	1.3±	0.78±	2.3±	1.9±	1.4±	3.1±	2.6±	1.4±	***	***	***
				0.30 ^abcde^	2.3 ^cdef^	1.9 ^f^	1.6 ^f^	3.0 ^f^	2.9 ^bcdef^	3.0 ^def^	2.2 ^ef^	0.49 ^ab^	0.18 ^a^	0.47 ^abc^	0.32 ^abc^	0.83 ^ab^	0.72 ^abcd^	0.28 ^abcd^	0.36 ^ab^			
P5	trans-neocnidilide	1755	B [19]	0.26±	0.24±	1.8±	0.16±	0.30±	0.78±	0.99±	0.94±	0.34±	0.13±	0.19±	0.08±	1.7±	0.59±	0.50±	0.24±	ns	ns	ns
				0.03	0.03	0.02	0.04	0.06	0.06	0.04	0.04	0.1	0.05	0.22	0.02	0.88	0.22	0.06	0.06			
P6	(E)-ligustilide	1764	B [32]	0.12±	0.14±	0.24±	0.23±	0.25±	0.14±	0.18±	0.18±	tr±	tr±	tr±	tr±	0.10±	tr±	tr±	tr±	***	ns	***
				0.02 ^abc^	0.10 ^abc^	0.01 ^c^	0.03 ^c^	0.05 ^c^	0.01 ^abc^	0.09 ^ab^	0.05 ^ab^	0.01 ^b^	0.01 ^b^	0.02 ^b^	0.01 ^b^	0.01 ^ab^	0.01 ^b^	0.01 ^b^	0.01 ^b^			
	Total			10	16	27	23	22	17	22	21	2.4	1.5	3.5	2.9	3.9	4.7	4.7	2.7			
	Oxides																					
O1	(Z)-limonene oxide	1147	A	nd ^a^	nd ^a^	nd ^a^	nd ^a^	nd ^a^	nd ^a^	nd ^a^	nd ^a^	nd ^a^	0.49±	0.87±	0.66±	1.1±	0.66±	1.7±	0.73±	***	***	***
													0.37 ^ab^	0.11 ^bc^	0.04 ^bc^	0.15 ^c^	0.05 ^bc^	0.26 ^d^	0.07 ^bc^			
O2	caryophyllene oxide	1610	A	tr±	0.13±	0.25±	0.10±	010±	0.10±	tr±	nd ^a^	nd ^a^	nd ^a^	nd ^a^	nd ^a^	nd ^a^	nd ^a^	nd ^a^	nd ^a^	***	***	***
				0.01 ^ab^	0.04 ^b^	0.05 ^c^	0.02 ^ab^	0.07 ^ab^	0.02 ^ab^	0.01 ^ab^												
	Total			0.04	0.13	0.25	0.05	0.08	0.09	0.02	0	0	0.49	0.87	0.66	1.1	0.66	1.7	0.73			
	Unknowns																					
U1	unknown 1	n/a		0.57±	0.31±	0.43±	0.19±	0.27±	0.71±	1.2±	0.51±	0.10 ±	tr±	tr±	tr±	0.11±	0.18±	0.13±	0.10±	***	**	***
				0.09 ^abc^	0.03 ^ab^	0.06 ^ab^	0.02 ^ab^	0.01 ^ab^	0.20 ^bc^	0.47 ^c^	0.29 ^abc^	0.02 ^ab^	0.02 ^a^	0.04 ^a^	0.01 ^a^	0.02 ^ab^	0.02 ^ab^	0.01 ^ab^	0.01 ^ab^			
U2	unknown 2	n/a		2.3±	1.7±	2.1±	0.84±	1.0±	2.7±	3.4±	1.5±	0.28±	0.22±	0.47±	0.14±	0.63±	0.65±	0.44±	0.24±	***	*	***
				0.63 ^abc^	0.03 ^abc^	0.06 ^abc^	0.02 ^ab^	0.01 ^ab^	0.20 ^bc^	0.47 ^c^	0.29 ^abc^	0.01 ^a^	0.05 ^a^	0.10 ^a^	0.04 ^a^	0.14 ^ab^	0.27 ^ab^	0.08 ^a^	0.05 ^a^			
U3	unknown 3	753		nd ^a^	nd ^a^	nd ^a^	nd ^a^	nd ^a^	nd ^a^	nd ^a^	nd ^a^	0.14±	tr±	tr±	nd ^a^	tr±	tr±	tr±	tr±	***	ns	***
												0.04 ^ab^	0.01 ^ab^	0.01 ^ab^		0.01 ^b^	0.01 ^ab^	0.01 ^a^	0.01 ^a^			
U4	unknown 4	1081		nd ^a^	nd ^a^	nd ^a^	nd ^a^	nd ^a^	nd ^a^	nd ^a^	nd ^a^	0.07 ±	tr±	0.10 ±	0.10 ±	0.10 ±	0.11±	0.15±	0.10 ±	***	***	***
												0.02 ^b^	0.02 ^b^	0.01 ^b^	0.02 ^b^	0.02 ^bc^	0.02 ^cd^	0.01 ^d^	0.01 ^bc^			
U5	unknown 5	1279		0.16±	0.10±	0.10±	0.13±	0.24 ±	0.11 ±	0.17 ±	0.10 ±	nd ^a^	nd ^a^	nd ^a^	nd ^a^	nd ^a^	nd ^a^	nd ^a^	nd ^a^	**	ns	**
				0.06 ^ab^	0.01 ^ab^	0.01 ^ab^	0.03 ^ab^	0.01 ^b^	0.01 ^ab^	0.03 ^ab^	0.04 ^ab^											
U6	unknown 6	1362		0.10±	0.10±	nd ^a^	0.16±	tr±	0.10±	0.10±	0.10±	nd ^a^	nd ^a^	nd ^a^	nd ^a^	nd ^a^	nd ^a^	nd ^a^	nd ^a^	***	*	***
				0.02 ^ab^	0.04 ^ab^		0.01 ^b^	0.04 ^a^	0.01 ^ab^	0.01 ^ab^	0.04 ^ab^											
U7	unknown 7	1539		0.25±	0.33±	0.19±	0.10 ±	0.15 ±	0.10±	0.18±	0.15±	nd ^a^	nd ^a^	nd ^a^	nd ^a^	nd ^a^	nd ^a^	nd ^a^	nd ^a^	***	*	***
				0.05 ^cd^	0.01 ^d^	0.02 ^bcd^	0.01 ^ab^	0.06 ^abc^	0.08 ^abc^	0.15 ^bcd^	0.06 ^abc^											
U8	unknown 8	1542		tr±	nd ^a^	0.10±	nd ^a^	0.10 ±	0.10±	0.10 ±	0.10 ±	nd ^a^	0.10±	0.10±	nd ^a^	0.10±	0.10±	tr±	0.11±	***	**	***
				0.01 ^a^		0.03 ^ab^		0.04 ^ab^	0.04 ^ab^	0.01 ^ab^	0.03 ^ab^		0.05 ^b^	0.02 ^b^		0.02 ^b^	0.02 ^ab^	0.01 ^ab^	0.01 ^b^			
U9	unknown 9	1653		nd ^a^	nd ^a^	nd ^a^	nd ^a^	nd ^a^	nd ^a^	nd ^a^	nd ^a^	0.10±	tr±	tr±	tr±	tr±	tr±	tr±	0.16±	**	**	**
												0.05 ^ab^	0.02 ^a^	0.02 ^a^	0.01 ^ab^	0.01 ^ab^	0.03 ^a^	0.01 ^ab^	0.08 ^b^			
U10	unknown 10	1776		nd ^a^	nd ^a^	nd ^a^	nd ^a^	nd ^a^	nd ^a^	nd ^a^	nd ^a^	0.04 ±	tr±	tr±	nd ^a^	tr±	tr±	tr±	tr±	***	ns	**
												0.02 ^ab^	0.01 ^ab^	0.01 ^ab^		0.02 ^ab^	0.03 ^ab^	0.01 ^ab^	0.01 ^ab^			
	Total			3.4	2.5	2.9	1.4	1.8	3.8	5.1	2.4	0.7	0.44	0.67	0.29	1	1.1	0.81	0.72			

^a^ Linear retention index on a HP-5MS column. ^b^ A, mass spectrum and LRI agree with those of authentic compounds; B, mass spectrum (spectral quality value >80 was used) and LRI agrees with reference spectrum in the NIST/EPA/NIH mass spectra database and LRI agree with those in the literature cited; ^$^ tentatively identified, spectral quality value of 70 was used for this compound. ^c^ Percentage composition of total peak area divided by compound peak area; means labelled with letters are significantly different (*p* < 0.05) according to the GxE interaction; means of three replicate samples; tr, trace amounts <0.10%; nd, not detected. ^d^ Probability, obtained by ANOVA, that there is a difference between means; ns, no significant difference between means (*p* > 0.05); * significant at the 5% level; ** significant at the 1% level; *** significant at 0.1% level. ^e^ Harvest year. ^f^ Genotype. ^g^ Harvest year × genotype interaction. Cells have been colour coded; red expresses the genotype with the higher value compared to harvest year; green expresses the genotype with the lower value compared to harvest year; no colour expresses no difference in percentage composition for both years.

Previous research has shown that monoterpenes comprise the majority of the aroma profile of celery. In this study and for both years, monoterpenes comprised the majority of the aroma composition of the eight celery genotypes, making up an average of 55% of the aroma composition in 2018 and 88% in 2020, which is a significantly higher proportion of the total profile and confirms previous research. Orav, Kailas and Jegorova [33] reported similar results in Estonian grown celery, where monoterpenes content comprised 85.3% of total flavour profile. In particular, limonene was one of the most abundant compounds with an average percentage composition of 31% in 2018 and 58% in 2020. Limonene odour has been described as citrusy, pine and minty [5,16]. These are not typical descriptors used to describe celery odour and although its prominence is dominant in celery, its contribution to the aroma profile is minimal. Other terpenoid compounds including camphene, α-pinene and β-pinene, γ-terpinene, β-caryophyllene, α-humulene and kessane identified in this study were also detected in many other studies in varying proportions [8,9,10,12,14,33,34].

Phthalide compounds are known as odour active compounds and main contributors to the characteristic odour of celery [2,15,33,34,35,36]. These compounds impart a “herbal” and “celery-like” aroma [5,16]. The proportion of the aroma profile comprised of phthalide compounds varied between years and genotype, with 2018 exhibiting a higher proportion composition compared to 2020. Lund, Wagner and Bryan [15] identified sedanenolide, 3-n-butylphthalide, hexahy-dro-3-n-butylphthalide and β-selinene to exhibit a celery-like odour. Three of these compounds were identified in all eight genotypes in both harvest years but their contribution to the composition varied. Sedanenolide and β-selinene had a higher proportion of the 2018 grown celery and are observed in the highest proportion in genotype 12. van Wassenhove, Dirinck, Vulsteke and Schamp [14] observed slight differences in the concentration of these compounds between years, however, unlike this study, no significant differences were reported. Furthermore, they presented a similar phthalide content, ranging from 6–11%, while in this study 19% and 3% was comprised of phthalides. The variation in the prominence of sedanenolide found in celery is very apparent not only in this study but in a plethora of studies where the percentage composition ranges from 0.2–39.5% [5]. Genotype 12 exhibited a high proportion of monoterpenes and the highest proportion of sesquiterpenes for both harvest years. In 2018, genotype 10 expressed the highest proportion of phthalides compared to other genotypes, exhibiting a high percentage of 3-n-butylphthalide (9.4%) and sedanenolide (15%) and genotype 12 had the highest proportion of sedanenolide (16%). On the other hand, genotypes 18 and 22 in 2020 exhibited the highest proportion of these compounds including 3-n-butylphthalide (3.1 and 2.6%, respectively). Turner et al. [5] identified 3-n-butylphthalide to be the most commonly reported phthalide [2,3,11,13,16,33,35,36]. Based on this observation, genotypes 10 and 12 in 2018 and genotype 22 in 2020 could be perceived as the genotypes with the strongest celery odour.

In terms of other compounds, smaller differences in the average composition between the years were observed: alcohols 1.3% and 0.15%, esters 0.16% and 0.5% and finally alkanes 1.6% for both 2018 and 2020 harvests, respectively. Limited research has been published about these types of compounds and their contribution to the celery aroma profile. By combining GC/MS and gas chromatography/olfactometry (GC/O), Turner et al. [16] identified compounds that contribute to the distinct celery aroma and how the aroma changed and developed throughout maturity. Using two of the same genotypes also used in this study (12 and 22), the aroma development over three time-points was studied: two-weeks before commercial maturity, at commercial maturity and two-weeks after commercial maturity. Monoterpene, sesquiterpene and phthalide compounds identified in the present study reflect those compounds observed by Turner et al. [16] and demonstrate that they are strongly influenced by maturity. Once commercial maturity was reached, the relative abundance of these compounds in the overall profile decreased, while alcohol and ester compounds became more abundant. Esters also identified by Turner et al. [16], including carveol acetate and hexyl hexanoate, were reported to contribute to green, herbal and damp odours in overmature celery according to GC/O analysis. The ester composition in the present study also varied as a consequence of both genotype and harvest year (Table 1) and a higher ester composition was observed from the 2020 harvest; however, methyl butanoate and (E)-pinocarvyl acetate were not significantly influenced by the genotype, only harvest year.

**Figure 1:**
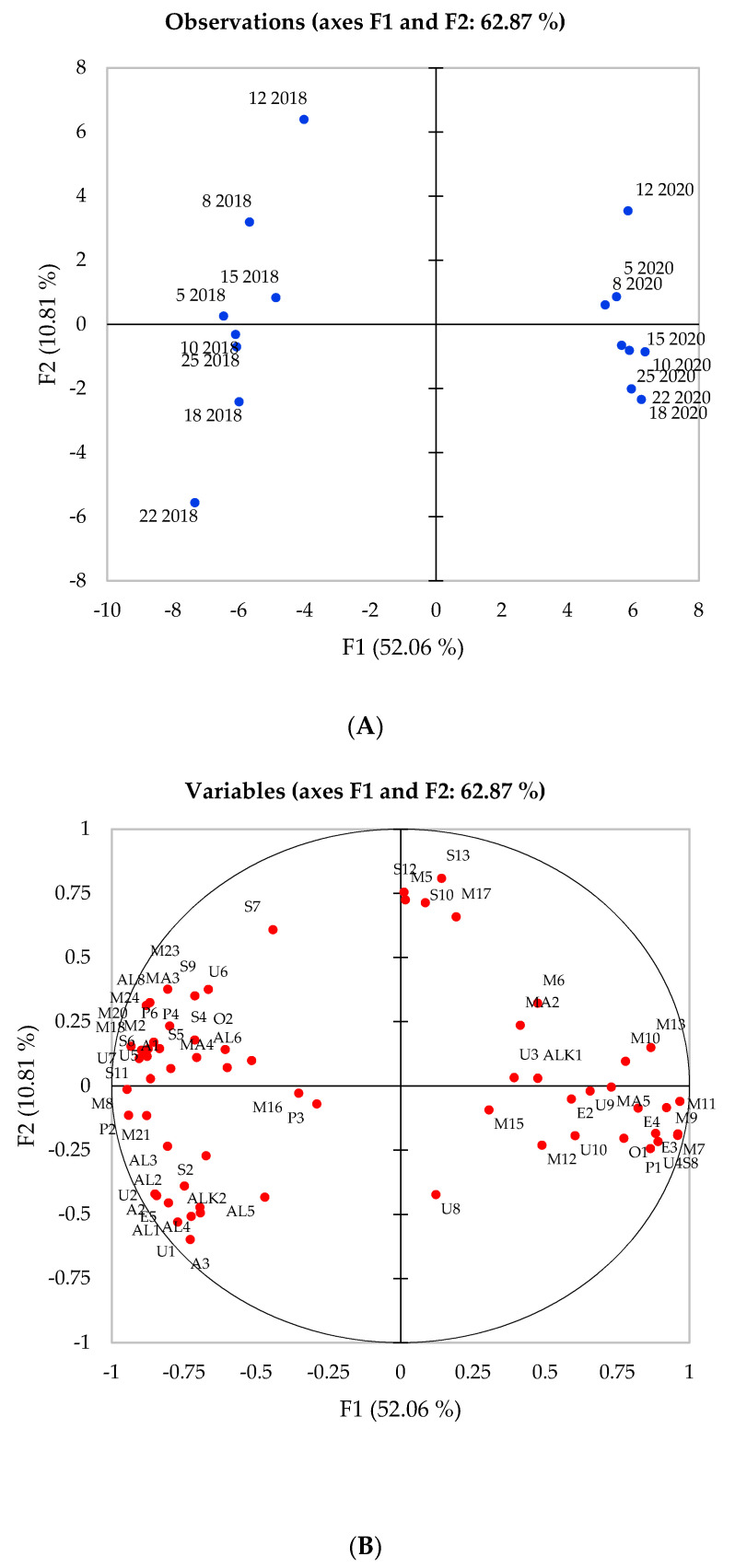

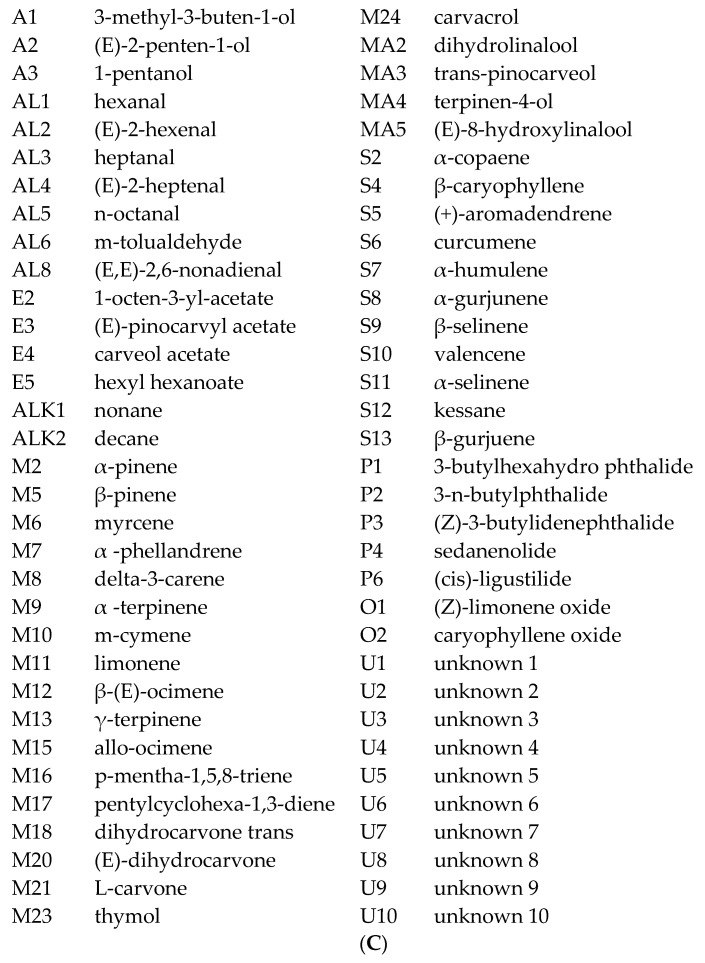
Principal component analysis of eight celery samples harvested in 2018 and 2020 showing correlations with volatile compounds. (**A**) Projection of the samples; (**B**) Distribution of variables; (**C**) Compound codes as appear in plot (**B**).

Principal component analysis (PCA) allowed for the visual comparison of the volatile composition of the eight celery genotypes in 2018 and 2020 (Figure 1) and the examination of any correlations occurring between genotype, harvest year and chemical compounds. Using only the significant compounds for harvest year, genotype and their interaction, a clear divide between the compounds associated with each year was observed. Principal component one (F1) and two (F2) explained 62.78% in total of the variation present in the data and it can be observed that the first axis separated samples from the two harvest years (2018 and 2020), while the second axis separated the various genotypes within a harvest year. Differences between the harvest years were apparent as is exhibited by the separation along the F1 component, which accounts for 52.06% of the variation. Genotypes were consistently separated across the F2 component for both years, which explains 10.81% of the variation. Metabolic pathways are genetically regulated, leading to the hypothesis that compounds that are important to a particular cultivar should remain fairly constant in their relative abundance between seasons and any deviations in these compounds are most likely due to external factors rather than genotype [37]. Genotypes 12, 8 and 5 for both years along with genotype 15 from 2018 were positively correlated with F2. Conversely, genotypes 10, 18, 22 and 25 for both years were negatively associated with F2.

Predominantly, monoterpenes and phthalides were separated across F2 and influenced by genotype, while sesquiterpenes, aldehydes and esters were separated across F1, respectively. Strong significant relationships were also observed between the compound groups, such as with alcohols and aldehydes expressing strong and positive correlations together, while low boiling monoterpenes including delta-3-carene and limonene expressed strong negative correlations with alcohols and aldehydes. Conversely, sesquiterpenes and phthalides had a negative correlation with the above monoterpenes and, instead, expressed a positive correlation with higher boiling monoterpenes including L-carvone, thymol and carvacrol.

In 2018, the genotype had a stronger influence over the volatile composition and this is reflected through the more noticeable separation between the eight genotypes and a stronger association with aroma compounds. However, genotypes 12, 18, 22 and 25 exhibited similar placement on the observation plot between the two years, albeit on opposing sides of F2. Monoterpenes (M2, 8, 16, 18, 21, 22, 23, 24), monoterpenoid alcohols (MA3, 4), sesquiterpenes (S2, 4, 5, 6, 9) and phthalides (P2, 3, 4,6) were positively correlated with celery samples grown in 2018. Conversely, monoterpenes (M6, 7, 9, 10, 11, 12, 13, 15), sesquiterpenes (S8, 10, 12, 13), monoterpenoid alcohols (MA2, 5) were positively correlated with celery samples grown in 2020. The spread of monoterpene and sesquiterpene compounds across the plot and presence within all genotypes across both years (Table 1) proves these are fundamental compounds to celery. As it can be observed from Figure 1, the aroma profile in 2018 consisted of a higher proportion of phthalide compounds than in 2020, where all phthalides, apart from 3-butylhexahydro phthalide (P1), appeared closely associated with the 2018 samples. Due to the odour active nature of sedanenolide and other phthalides and the strong celery odours that these compounds impart, celery genotypes exhibiting a high proportion of these compounds are more likely to possess a strong characteristic celery odour.

The harvest year and genotype both had an influence on the volatile content of celery samples, however, a much stronger influence over the percentage composition for all genotypes and the majority of volatile compounds was observed by harvest year. Genotypes exhibited fewer significant differences over the majority of monoterpenes, aldehydes, sesquiterpenes and phthalides. Although the genotype is known to play a role in predetermining the aroma composition [37], the variation caused by harvest year and, therefore, the growing environment possessed a more significant role in determining the aroma composition (Table 1, Figure 1). Differences in climate during growth are most likely the cause of these compositional changes and will be discussed further in Section 3.3. The aroma and flavour quality of certain genotypes such as 12, 18 and 25 were consistent across the two years demonstrating that these genotypes may provide consistent quality crop for celery growers and breeders irrespective of the environmental changes. Carrying out sensory profiling on these cultivars will permit the examination of the impact of the different compositions caused by genotype and harvest year on flavour perception.

### 3.2. Sensory Evaluation of Fresh Celery Samples

The sensory profile of the eight celery samples was generated by a trained panel who came to the consensus of 22 and 24 terms for the quantitative assessment of samples in the 2018 and 2020 samples, respectively. The two additional attributes in 2020 were that of “fresh parsley flavour” and “celery residue in mouth” as an aftereffect. Table 2 shows the mean panel scores for these attributes. Out of the 22 attributes that were profiled in 2018, 14 of these were found to be significantly different between the genotypes and in 2020, 18 out of the 24 attributes were found to be significantly different. There were few significant assessor × sample interactions identified for both the 2018 and 2020 harvests, which suggests that the panelists scored samples in a consistent manner [38].

Statistical comparison of sensory differences between years could not be completed due to the two-year difference between harvests, however, general trends will be discussed. All appearance attributes showed a strong significant difference for both years between genotypes and this is due to the fact that the genotypes selected for the study included genotypes that were white, green or pink and with varying heights. The scoring for these attributes remained consistent between years for each genotype. Similarly, mouthfeel attributes of crunchiness and moistness scored consistently between the years for each genotype. A relationship between the ribbed appearance of the petiole with the stringiness mouthfeel was observed and it changed significantly between the years for individual genotypes more than any other attribute. Lignin, a key component in providing mechanical structure in higher plants, such as celery, has been shown to be influenced by abiotic and biotic stresses. Low temperatures have been observed [39,40] to influence the synthesis of lignin and its precursors. Li et al. [41] identified all microRNAs two celery varieties to be sensitive to temperature stress and a stronger response was observed towards cold stress, suggesting that cooler temperatures are optimal for celery growth. The structural differences observed in the genotypes in 2018 could be a response to stress and the cooler temperatures of 2020 provided optimal temperatures for lignin synthesis, which causes these genotypes to be perceived as more crunchy, stringy and firm.

**Figure 2:**
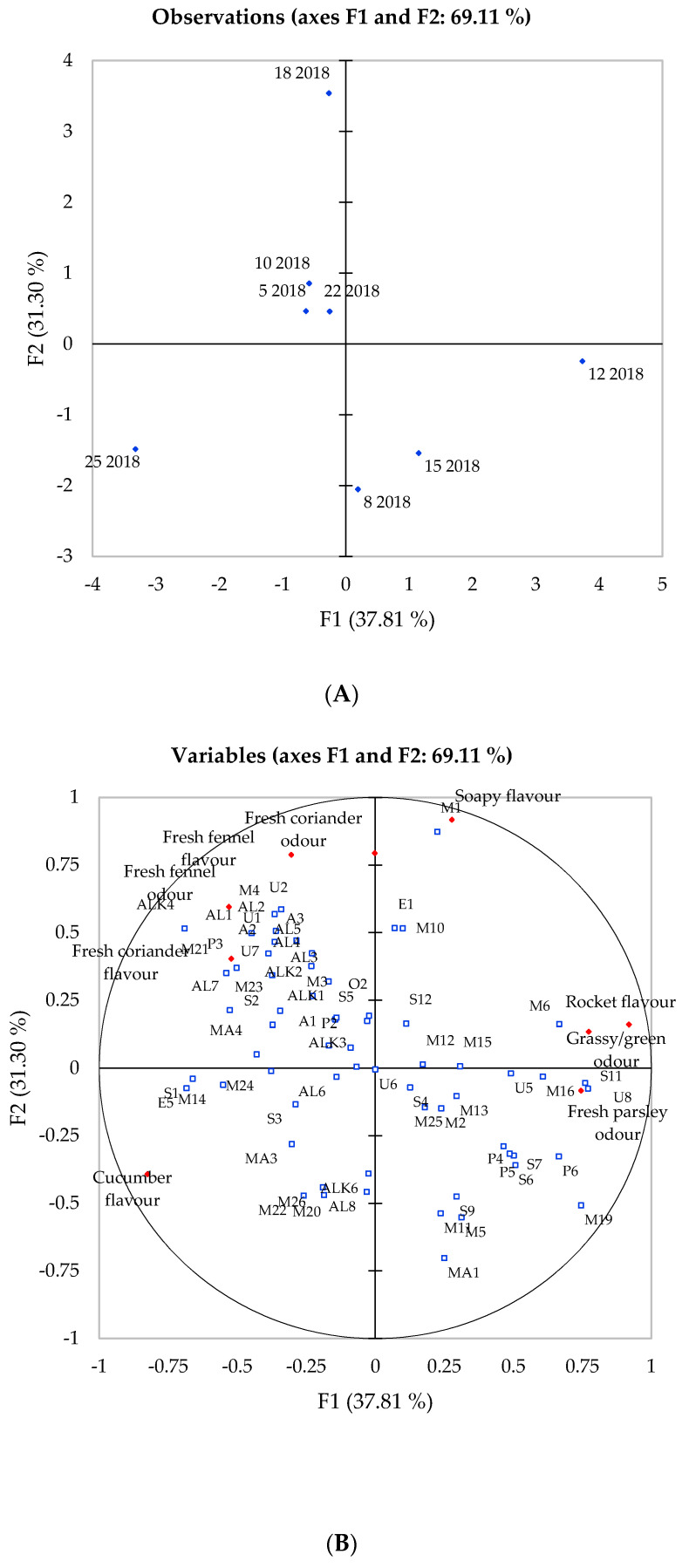

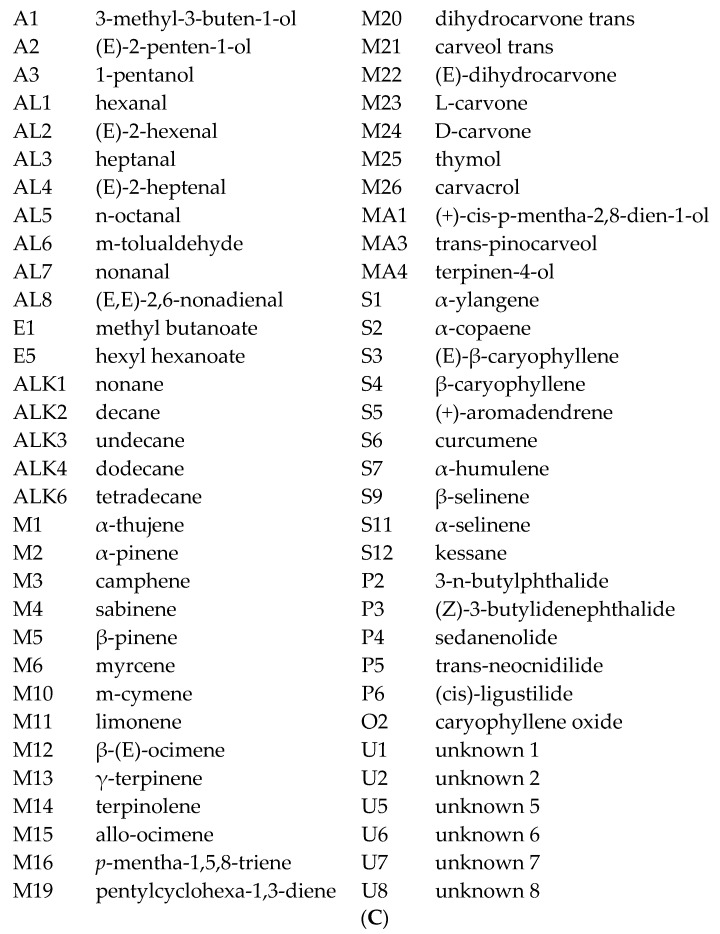
Principal component analysis of eight celery samples harvested in 2018 showing correlations with volatile compounds and sensory attributes. (**A**) Projection of the samples; (**B**) Distribution of variables; (**C**) Compound codes as they appear in plot (**B**).

**Figure 3:**
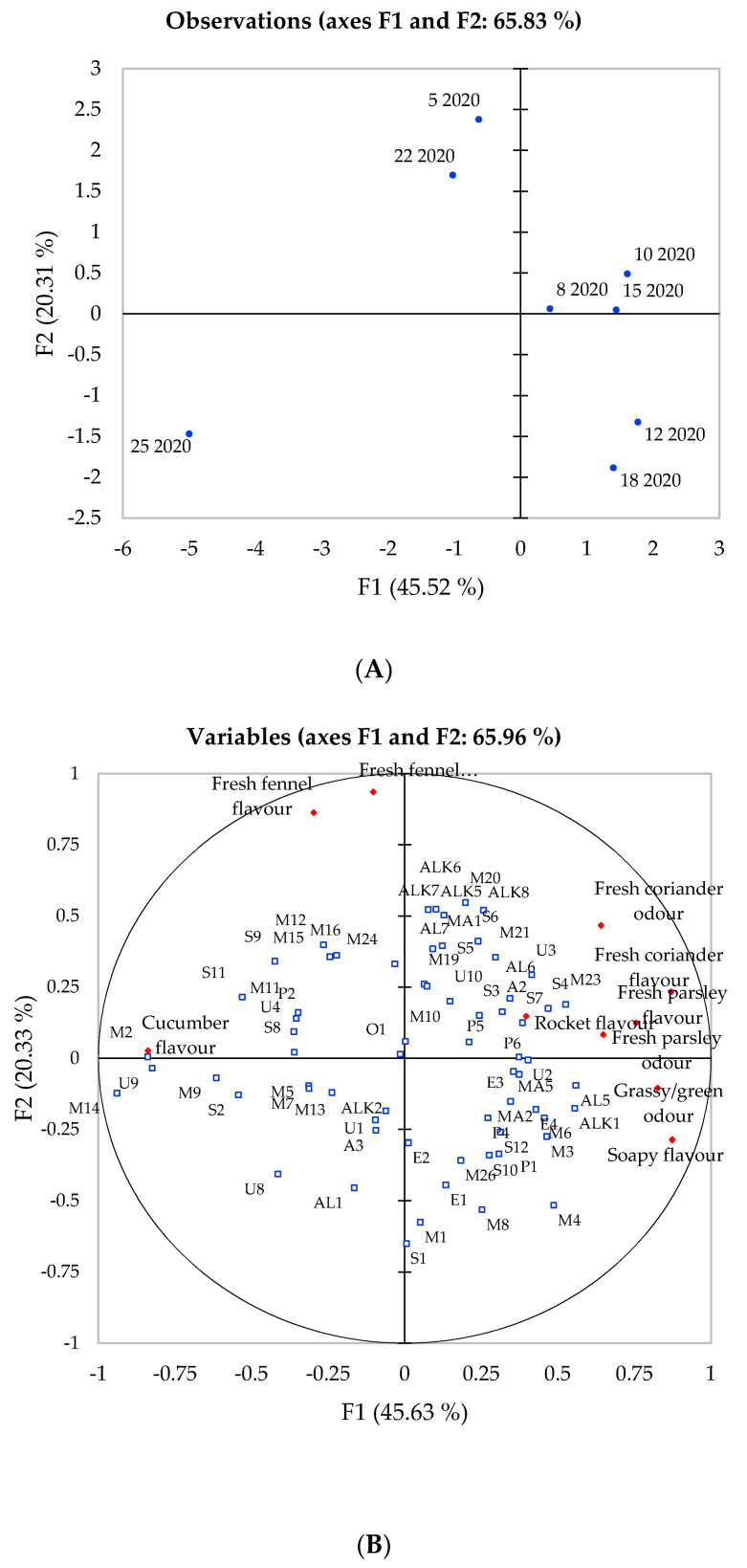

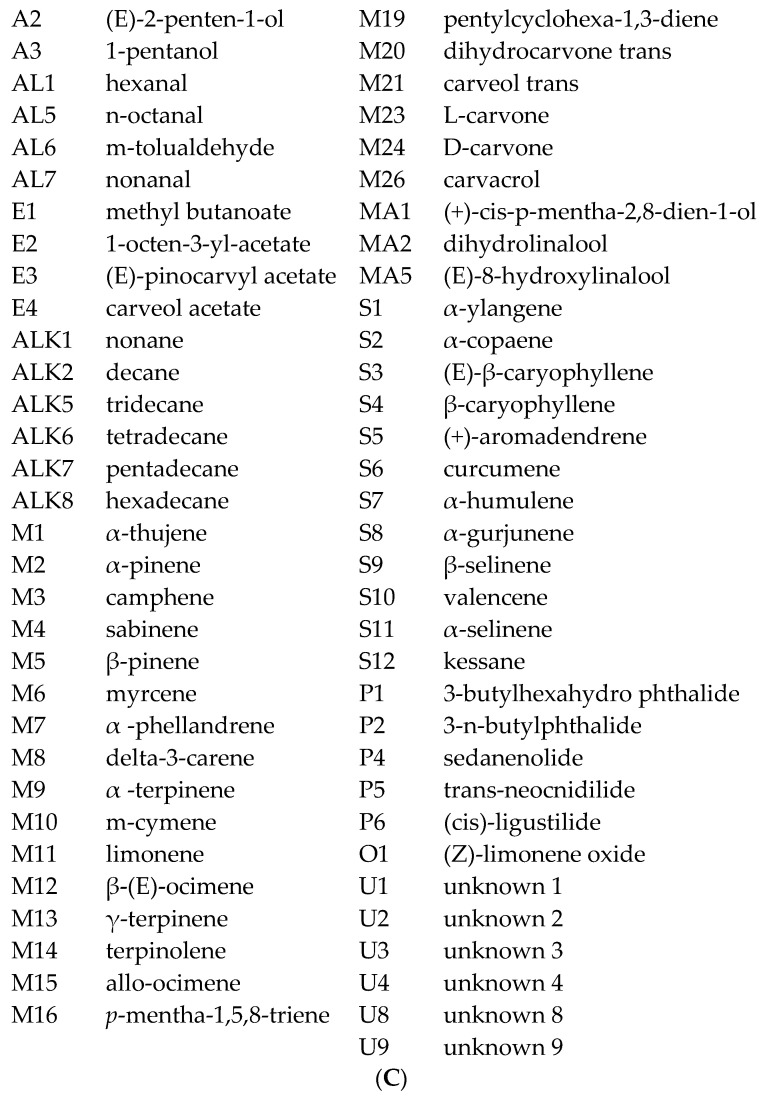
Principal component analysis of eight celery samples harvested in 2020 showing correlations with volatile compounds and sensory attributes. (**A**) Projection of the samples; (**B**) Distribution of the variables; (**C**) Compound codes as they appear in plot (**B**).

The odour and flavour attributes evaluated displayed clear significant differences between both genotypes and harvest year. The attributes “watery/cucumber” and “rocket” flavour along with “grass/green” odour were scored highly in the 2018 harvest, while “fresh fennel and parsley” flavour were scored highly in the 2020 harvest. “Fresh coriander” aroma and flavour along with “soapy” flavour were scored similarly for both years. Genotype 25 was scored low for both years for flavour and aroma attributes apart from the “watery/cucumber” flavour, while genotype 12 was scored as the most bitter for both years. Combining these attributes with the volatile compounds identified through GC/MS (Table 1) provided a deeper understanding in the differences within the aroma composition and its impact on flavour perception. Principal component analysis was used to visualise the sensory and chemical differences across the eight genotypes and the volatile compounds identified (Table 1) and the attributes related to odour and flavour were used as variables (Figure 2 and Figure 3).

Firstly, a clear variation between the genotype was observed in 2018 (Figure 2) whereby principal component one (F1) and two (F2) explained 69.11% of the total variation within the data. The first axis separates genotypes 5, 10, 18 and 22 from other genotypes, whereas the second axis separates genotypes 8, 15 and 12. Genotype 25 had low scores for most of the flavour attributes and only scored high in the watery and cucumber flavour. On the other hand, genotype 12 negatively correlated with genotype 25 and was associated with a parsley and grass-like odour with a rocket aftertaste. Genotype 18 was positively correlated to the fresh fennel flavour with the soapy characteristics that accompany many members of the Apiaceae family, such as coriander. A grouping of aroma compounds in the centre of the PCA was observed, whereas the sensory characteristics were positioned in the outer rim of the biplot with genotypes 5, 10 and 22 grouped in the middle of the observation plot. Apart from genotype 10, these exhibited an average volatile content (Table 1) compared to genotype 12 along with no strong association with sensory attributes (Figure 2). Many of the phthalides were associated with genotypes 12 and 10.

**Table 2:** Mean panel scores for sensory attributes of the eight celery samples harvested in 2018 and 2020.

Attribute	Score ^A^																	
	2018									2020								
	5	8	10	12	15	18	22	25	*p* ^b^	5	8	10	12	15	18	22	25	*p* ^B^
Appearance																		
Colour	56.4 ^b^	63.6 ^ab^	62.6 ^ab^	72.9 ^a^	72.1 ^a^	65.6 ^ab^	70.5 ^a^	26.8 ^c^	***	46.3 ^cd^	53.0 ^bcd^	44.6 ^d^	67.5 ^ab^	61.0 ^abc^	55.6 ^abcd^	70.5 ^a^	14.7 ^e^	***
Stalk thickness	49.8 ^ab^	49.5 ^ab^	55.8 ^a^	20.9 ^b^	58.7 ^a^	62.5 ^a^	61.3 ^a^	55.0 ^a^	***	60.6 ^abc^	47.7 ^cde^	36.2 ^def^	20.7 ^ee^	51.1 ^cd^	74.1 ^a^	72.0 ^ab^	59.8 ^abc^	***
Ribbed	46.6 ^bc^	61.0 ^ab^	61.7 ^a^	65.9 ^a^	35.5 ^cd^	25.4 ^d^	34.2 ^cd^	37.4 ^cd^	***	60.3 ^ab^	65.8 ^a^	66.6 ^a^	68.5 ^a^	45.9 ^b^	50.7 ^b^	56.4 ^ab^	55.6 ^ab^	***
Aroma																		
Fresh fennel	16.5	14.2	18.9	15.5	15.3	18.6	15.4	18.2	ns	32.1	22.1	22.8	21.1	23.6	19.8	30.8	20.3	*
Grassy/green	32.6 ^a^	31.0 ^ab^	32.1 ^ab^	36.3 ^a^	30.7 ^ab^	28.3 ^ab^	35.3 ^a^	21.1 ^b^	***	27.1 ^ab^	33.8 ^a^	25.9 ^ab^	32.8 ^a^	34.5 ^a^	34.6 ^a^	28.5 ^ab^	18.2 ^b^	***
Fresh parsley	14.1	19.7	19.0	19.1	20.6	16.7	16.7	10.8	ns	18.0	19.2	20.8	16.8	20.6	19.4	17.3	16.4	ns
Fresh coriander	12.8	12.1	14.2	11.7	14.2	17.5	15.4	11.1	ns	15.4	13.0	14.8	12.0	14.2	16.6	16.3	7.7	ns
Taste/flavour																		
Bitter	23.1 ^abc^	24.0 ^abc^	24.7 ^abc^	35.9 ^a^	28.2 ^abc^	31.3 ^ab^	24.4 ^abc^	15.5 ^c^	**	33.2 ^abc^	20.6 ^abc^	35.0 ^ab^	38.4 ^a^	35.2 ^a^	34.4 ^ab^	33.0 ^abc^	19.6 ^c^	***
Sweet	15.2 ^bcd^	20.3 ^ab^	21.6 ^ab^	10.6 ^d^	15.6 ^bcd^	12.2 ^cd^	20.0 ^ab^	24.6 ^a^	***	17.3 ^abc^	25.0 ^abc^	20.0 ^abc^	17.1 ^abc^	13.1 ^c^	14.8 ^bc^	18.1 ^abc^	23.7 ^ab^	**
Fresh fennel	11.9	10.3	12.6	11.0	7.7	13.6	11.6	11.3	ns	27.5 ^a^	23.5 ^ab^	23.3 ^ab^	16.9 ^ab^	21.1 ^ab^	13.7 ^b^	23.3 ^ab^	21.3 ^ab^	**
Rocket	11.3 ^bc^	13.4 ^bc^	12.4 ^bc^	23.8 ^a^	16.6 ^abc^	16.9 ^abc^	10.4 ^bc^	7.7 ^c^	***	1.1	1.8	2.7	3.8	4.2	0.7	3.4	1.3	ns
Fresh coriander	17.5	16.3	16.0	9.6	15.0	18.1	18.9	14.1	ns	17.2	18.2	21.2	19.1	16.7	18.2	17.9	11.6	ns
Soapy	18.2 ^ab^	12.4 ^b^	16.4 ^ab^	18.4 ^ab^	15.4 ^ab^	23.7 ^a^	16.3 ^ab^	13.0 ^ab^	*	14.9 ^ab^	14.2 ^ab^	19.1 ^ab^	20.0 ^a^	17.4 ^ab^	22.9 ^a^	14.1 ^ab^	9.3 ^b^	***
Watery/cucumber	25.7 ^ab^	33.2 ^ab^	30.4 ^ab^	9.1 ^c^	30.0 ^ab^	22.4 ^b^	27.9 ^ab^	37.7 ^a^	***	19.8 ^ab^	15.7 ^ab^	12.1 ^b^	10.8 ^b^	16.2 ^ab^	20.5 ^ab^	23.2 ^ab^	27.0 ^a^	**
Fresh parsley	nd	nd	nd	nd	nd	nd	nd	nd		15.5	14.7	13.8	16.7	15.2	13.0	11.0	9.7	ns
Mouthfeel																		
Crunchy	65.4 ^abc^	62.6 ^bc^	64.9 ^abc^	56.7 ^c^	70.2 ^ab^	66.4 ^abc^	73.7 ^a^	62.5 ^bc^	***	70.6 ^ab^	65.8 ^ab^	72.9 ^a^	66.7 ^ab^	74.2 ^a^	58.5 ^b^	74.7 ^a^	67.6 ^ab^	**
Stringy	40.8 ^b^	46.6 ^b^	40.1 ^b^	64.1 ^a^	33.2 ^b^	40.6 ^b^	35.1 ^b^	35.2 ^b^	***	53.2 ^bc^	62.8 ^ab^	61.8 ^ab^	74.2 ^a^	54.4 ^bc^	45.7 ^c^	51.1 ^bc^	45.1 ^c^	***
Moist	50.6 ^a^	47.2 ^a^	50.0 ^a^	29.7 ^b^	53.1 ^a^	44.3 ^a^	51.4 ^a^	54.8 ^a^	***	55.0 ^abc^	51.0 ^bc^	44.8 ^c^	28.3 ^d^	49.3 ^bc^	50.3 ^bc^	54.8 ^bc^	57.6 ^ab^	***
Firmness of first bite	63.7	59.9	63.3	59.2	68.9	65.7	67.6	58.6	ns	69.3 ^ab^	65.2 ^ab^	68.1 ^ab^	66.2 ^ab^	72.4 ^ab^	60.6 ^b^	74.9 ^a^	65.1 ^ab^	*
After effects																		
Celery residue in mouth	nd	nd	nd	nd	nd	nd	nd	nd		51.4 ^ab^	51.1 ^ab^	52.5 ^ab^	64.0 ^a^	48.3 ^b^	45.8 ^b^	48.8 ^ab^	39.4 ^b^	***
Soapy	16.9 ^ab^	15.7 ^ab^	16.7 ^ab^	21.2 ^ab^	19.9 ^ab^	24.8 ^a^	18.6 ^ab^	12.9 ^b^	*	15.4 ^b^	14.4 ^b^	21.1 ^b^	23.2 ^a^	18.0 ^b^	21.2 ^b^	14.4 ^b^	14.6 ^b^	**
Grassy/green	27.7	27.0	27.9	27.6	28.4	26.4	31.4	19.0	ns	14.8	20.6	19.0	18.4	21.3	20.1	21.7	15.3	ns
Numbness	13.1	8.6	9.6	11.5	10.0	14.0	9.8	9.0	ns	11.4 ^a^	12.1 ^a^	11.5 ^a^	11.7 ^a^	12.6 ^a^	13.2 ^a^	9.8 ^b^	7.3 ^b^	**
Bitter	17.4 ^bc^	18.4 ^bc^	18.3 ^bc^	29.0 ^a^	19.1 ^bc^	25.7 ^ab^	16.0 ^bc^	12.0 ^c^	***	18.0 ^bc^	20.9 ^abc^	28.5 ^a^	27.5 ^ab^	25.5 ^ab^	23.0 ^abc^	19.6 ^abc^	13.5 ^c^	***

^A^ Means are from two replicate samples; differing small letters (a, b, c, d, e, f) represent sample significance from multiple comparisons and means not labelled with the same letters are significantly different (*p* < 0.05); nd, not detected. ^B^ Probability obtained by ANOVA that there is a difference between means; ns, no significant difference between means (*p* > 0.05); * significant at the 5% level; ** significant at the 1% level; *** significant at 0.1% level.

Overall, it seems that the majority of monoterpenes were negatively correlated with the first principal component (F1) and compounds belonging to classes such as alcohols, sesquiterpenes and phthalides were positively associated with F1 along with the majority of the flavour attributes. Samples harvested in 2018 exhibited a lower proportion of monoterpenes but a higher proportion of alcohols and aldehydes, thus, explaining the low association with many of the flavour and aroma attributes from the sensory analysis.

In 2020, principal component one (F1) and two (F2) explained 65.96% of the total variation present and it can be observed that the first axis separates genotypes 5, 8, 10, 15 and 22, whereas the second axis separates genotypes 12, 18 and 25. According to the data presented in Figure 3, the genotype appears to express a weaker influence over the volatile composition than in 2018, which explains 20.31% of the variation present within the data. Differences in the volatile composition for the celery samples harvested in 2020 resulted in differences in the flavour perception. Compared to 2018 where genotypes 12, 18 and 25 were reported as the most distinctive, genotypes 5, 10, 12, 18, 22 and 25 became more distinguished from the remainder genotypes and displayed close associations with individual attributes. “Fresh fennel” was shown to be closely associated with genotype 18 in 2018, but became more strongly associated with genotypes 5 and 22 in 2020. In 2020, “fresh coriander”, “parsley” and “grass green” positively correlated with F1 were associated with genotypes 8, 10, 12, 15 and 18, while the “fresh fennel” odour and flavour attributes in the top left quadrant (Figure 3) were associated with genotypes 5 and 22. The cucumber flavour remained in a similar position for both years, showing a close association to genotype 25. The most consistent genotype out of the eight was genotype 25 in terms of sensory and volatile profile; in both harvests, it appeared to be the least aromatic reflected by its close association to the cucumber flavour. Celery samples harvested in 2020 exhibited a higher proportion of monoterpenes which contribute to the herbal sensory attributes. Within the correlation matrix, fresh fennel exhibited many positive correlations with compounds that contribute to warm, herbal, sweet and spearmint odours such as (E)-dihydrocarvone (M20), L-carvone (M24), (E)-β-caryophyllene (S3) and α-humulene (S7) as well as sedanenolide (P4) and (cis)-ligustilide (P6). Afifi, El-Mahis, Heiss and Farag [42] classified 12 fennel varieties based on their aroma profile and similarities can be observed when comparing the monoterpene profile of celery in this study with the aroma profiles of the fresh fennel used by Afifi et al. [42].

According to the results presented so far, samples harvested in 2020 had a more complex aroma profile leading to more flavourful genotypes compared to those harvested in 2018. Genotypes such as 10, 12 and 15 had a strong association with odour active compounds such as phthalides and, thus, associated with herbal flavour attributes such as fennel, coriander and parsley. However, genotypes grown in 2018 expressed a higher proportion of phthalides, which suggests that the typical celery odour would be more noticeable in these celery genotypes. Thappa et al. [43] investigated the variation of major components of genetically improved celery and reported that celery with a high phthalide content, such as those harvested in 2018, led to higher quality celery. The confirmation of whether this statement remains true for the celery used in this study requires the completion of consumer acceptability and preference trials.

### 3.3. Environmental Differences between Harvest Years and Influence on the Aroma Profile

In this study, clear differences in the volatile and sensory profile of the same genotypes grown in the same region of the United Kingdom across two different years were observed. Environmental data including climatic variances in temperature, rainfall and relative humidity were collected at the nearest weather station to the farm of growth and provided by G’s Fresh (Table 3). These environmental differences were hypothesised to influence the chemical composition within the crop. The daily air temperatures in 2018 (average 18 °C) were much higher than those in 2020 (average 14 °C). This change in temperature may have led to a warmer soil temperature in 2018, with a daily average presented to be over 7 °C warmer than in 2020. Although no differences in the volume of precipitation between years were observed, a large difference can be seen between the relative humidity. The impact of different growing conditions, such as temperature, on the flavour composition in celery is inadequately investigated and, within this experiment, only two growing seasons have been used; therefore, any conclusions that are drawn here can only be hypothesised. The utilisation of multiple years would generate more data and information about how celery responds to different climates and environments, which would produce a robust and vast dataset that will indicate more significant relationships between the plant’s response towards the environment and confirm or disprove any of the theories discussed in this section.

Being such a widely grown and consumed crop, it was expected that certain celery cultivars have been developed to grow under a range of temperatures. For example, cultivars EC 99249-1, RRL 85-1 and NRCSS-A have been identified as suitable for growth under the Indian climate, producing excellent essential oil content and high yield [44,45]. However, climates with long growing seasons with temperatures between 16 °C and 21 °C, with light rainfall and suitable irrigation, are thought to be optimal growing conditions for celery [6]. Kader [46] identified that preharvest factors including environmental conditions (temperatures, rainfall and wind speed) and agricultural techniques (planting density, irrigation and pesticide regimes) could often result in a decline in flavour quality. For other crops, such as apples, that are dependent on ester formation for flavour, Fellman, Miller and Mattinson [37] stressed the importance of genotype along with abiotic factors such as growing temperatures and cultural practices and they stated that these are “critical factors” involved in the synthesis of precursors involved in ester formation. Esters comprised a higher proportion of the aroma profile of celery grown in 2020 than celery grown in 2018 (Table 1), contributing to aroma such as fruity, apple and green and are shown to be associated with a grassy/green odour (Figure 3). With respect to celery, perhaps the lower temperatures exhibited in 2020 were more preferable for ester formation.

The influence of temperature on isoprene formation, the smallest terpene unit and building block for more complex monoterpenes, has been discussed by Sharkey, Wiberley and Donohue [47], whereby isoprene expresses a relationship with temperature and light and provides plant protection in the form of thermotolerance. Light and temperature have an influence in controlling the monoterpene and sesquiterpene plant emission as reported by Ibrahim et al. [48], where the total monoterpene and sesquiterpene emissions in silver birch (*Betula pendula*) and European aspen (*Populus tremula*) trees increased at higher temperatures and peaked at 18 °C. Sesquiterpene content was positively correlated to temperature whilst monoterpenes expressed the opposite and was identified at higher abundances at lower temperatures. These findings support the volatile results from celery presented in Table 1, where the total sesquiterpene content was higher in 2018 when higher temperatures were recorded and, by contrast, monoterpenes comprised the majority of the aroma profile in 2020 when lower temperatures were observed. From these findings it can be hypothesised that sesquiterpenes act as a protective mechanism from heat stress within celery.

How phthalide compounds, the characteristic compounds imparting celery odour, react to different environmental stimuli have not previously been studied. Although existing research discusses the importance of their presence in celery samples, there is a poor understanding on how they are synthesised and what the factors that influence the abundance of these compounds are [5]. Sedanenolide made up the highest proportion of the phthalide profile in both 2018 and 2020, albeit much higher in 2018. Overall, samples harvested in 2018 had a higher total phthalide content than celery grown in 2020, which mimicks a similar pattern to sesquiterpenoid compounds (Table 1) and, thus, possibly acts as a protective mechanism in response to the heat stress. Synthesising aromatic compounds is a standard response to abiotic stresses, such as temperature, in order to protect the crop [49]. Possessing a lower total phthalide content in 2020 explained why aromas and flavours such as fresh coriander and parsley were revealed and are becoming more apparent to human assessors (Table 2).

## 4. Conclusions

Harvest year showed a stronger influence over the aroma composition of eight celery genotypes compared to genotypes, leading to differences in the aroma profile and, thus, creating sensory differences between two different years. Completing volatile analysis and sensory evaluation of the eight genotypes of celery demonstrated that the celery genotypes harvested in 2018 were perceived as being less herbal and associated with green aroma and cucumber flavour compared to the samples harvested in 2020. Samples harvested in 2020 imparted herbal flavour notes such as parsley, fennel and coriander, which are all members of the Apiaceace family potentially because these flavour notes were revealed when dominant aromas derived from pthalides were less abundant.

Although the genotypes were observed to play less of a role than the harvest year, the genetic make-up of the crop undoubtedly plays a role in predetermining the flavour profile as well as the capacity to synthesise aroma compounds in response to stress [37,46,47,48], as shown by a high proportion of compounds expressing significant differences according to genotype, the variation caused by genotype and the variation in genotype perception from sensory evaluation. The eight genotypes used in this study all exhibited clear differences within the aroma composition; however, less variation between years was apparent for genotype 25, which imparted a cucumber flavour and was less associated with aromatic compounds. Similarly genotype 12, with a strong fresh parsley odour, had a constant aroma profile over the two harvest years and expressed a high proportion of sesquiterpenes and phthalide compounds according to the volatile composition.

The influence of the environment on the aroma composition was also evident in this study (Figure 1) with the majority of the compounds identified as significantly different between the two harvest years. The chemical composition was different in each year, with alcohol (including monoterpenoid alcohols), aldehyde, sesquiterpene and phthalide content all being in higher proportions in 2018. The warmer and dryer climates experienced in 2018 could explain these compositional differences, particularly with sesquiterpene and phthalide compounds, which have been previously observed to act as a crop protective mechanism in response to heat stress. Taking into consideration these observations, the celery grown in 2018 could be the preferred flavour, but this hypothesis would require consumer acceptability and preference trials to confirm this.

There is currently limited research to support the impact of the environment on the volatile composition and sensory profile of celery and, in order to confirm the environmental role, further work using controlled growth combined with sensory and chemical analysis needs to be carried out to provide a deeper understanding of the environmental relationship and how it affects volatile composition. Additionally, growing celery in alternative geographical locations could elucidate this relationship and provide more evidence as to how different environments affect the volatile composition. Providing explanations concerning the causes of aroma composition variation within celery, as well as other Apiaceae crops, will aid breeders to focus breeding programs on temperature resistant crops or steer fresh produce growers to utilise crops that are more resilient to the geographical climate of growth. These considerations, combined with regular inhouse taste panels and quality testing, will ultimately lead to better tasting crops with more stable flavour qualities.

## Figures and Tables

**Table 3:** Environmental data recorded at the nearest weather station to the farm of celery growth and provided by G’s Fresh.

Weeks after Field Transplant	2018				2020			
	Air Temp (°C)	Soil Temp (°C)	Rainfall (mm)	Relative Humidity (%)	Air Temp (°C)	Soil Temp (°C)	Rainfall (mm)	Relative Humidity (%)
1	17.0	17.1	0.0	73.0	9.8	9.6	0.1	82.0
2	14.7	17.3	0.0	81.3	11.4	10.7	0.0	74.6
3	16.4	18.1	0.1	66.1	9.4	9.9	0.0	67.9
4	17.0	24.4	0.0	94.8	16.7	16.9	0.0	63.3
5	18.9	27.9	0.0	98.5	15.7	17.3	0.0	62.3
6	19.8	28.6	0.0	99.7	14.4	16.1	0.0	71.1
7	18.2	25.5	0.0	99.4	12.0	12.6	0.0	86.4
8	20.4	29.0	0.0	99.0	17.2	18.3	0.2	80.7
9	21.4	26.7	0.1	70.5	19.6	21.5	0.0	69.1
10	20.9	27.7	0.0	71.8	16.0	18.6	0.0	78.9
11	17.3	20.7	0.2	99.9	16.0	17.6	0.2	86.6
12	18.4	28.6	0.0	98.6				
13	15.8	17.5	0.0	93.9				
Average	18.2	23.8	0.2	88.1	14.3	15.4	0.05	74.8

## Data Availability

The data presented in this study is available upon request from the corresponding author.

## Supplementary Materials

The following are available online at https://www.mdpi.com/article/10.3390/foods10061335/s1, Table S1: Origin and images of the eight celery samples used in this study and harvested in 2018 and 2020.
