# Supplementary material for: Investigating the Relationship of Genotype and Climate Conditions on the Volatile Composition and Sensory Profile of Celery (Apium graveolens)
Source: Foods. 2021 Jun 10;10(6):1335. doi: 10.3390/foods10061335 (PMC8227241; doi:10.3390/foods10061335)
Supplement: Supplementary file 1 [file foods-10-01335-s001.zip › foods-1246887-supplementary.pdf]

Table S1: Origin and images of the eight celery samples used in this study and harvested in 2018 and 2020.

Line	Origin	Harvest 2018	Harvest 2020
5	USA	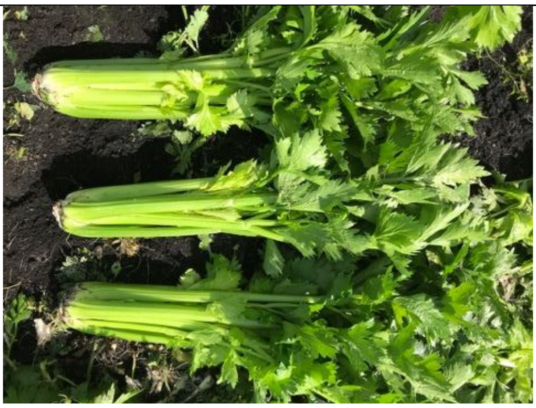	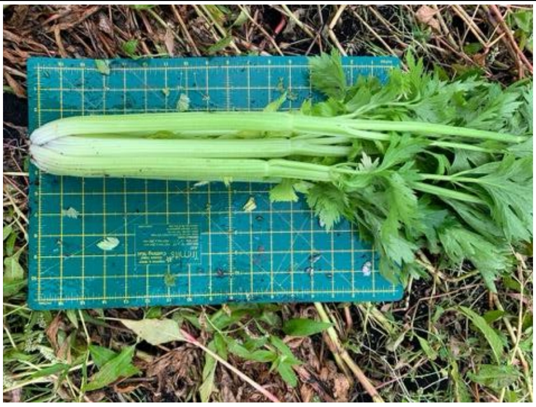
8	AUS	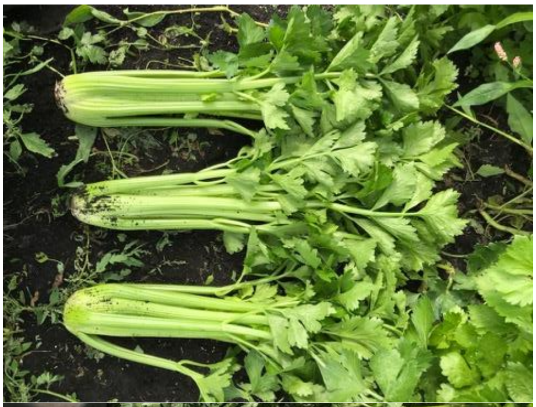	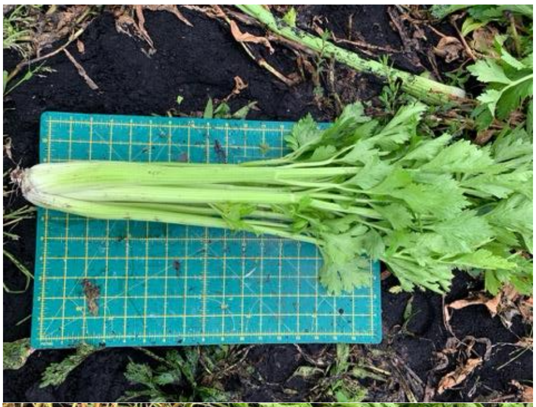
10	UK	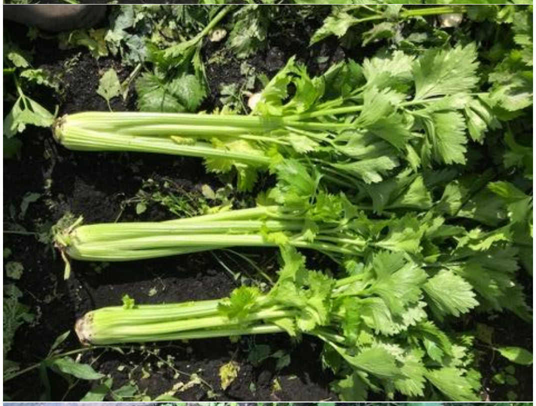	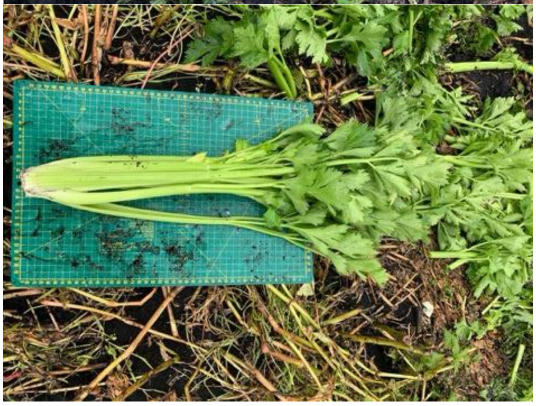
12	UK	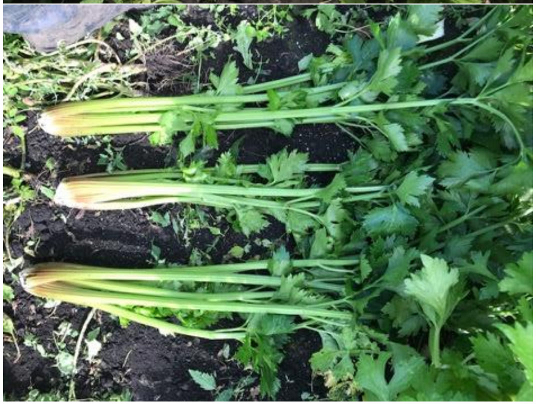	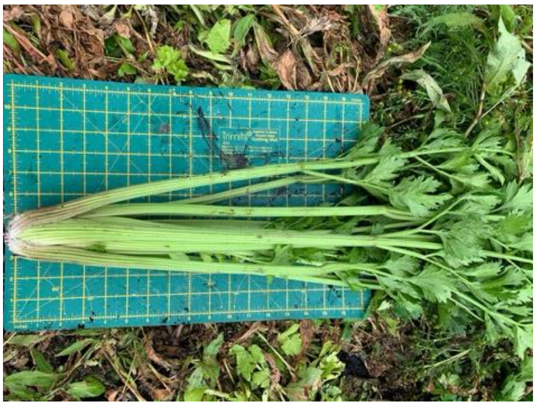

15	USA	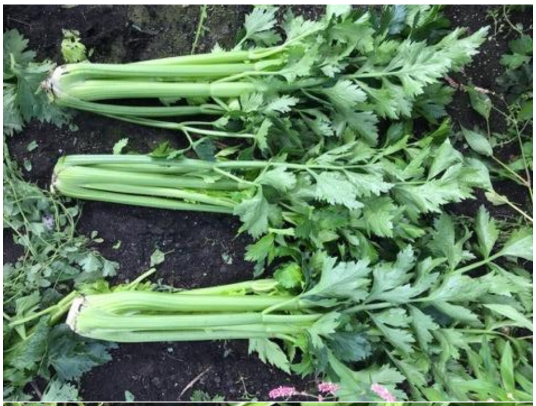	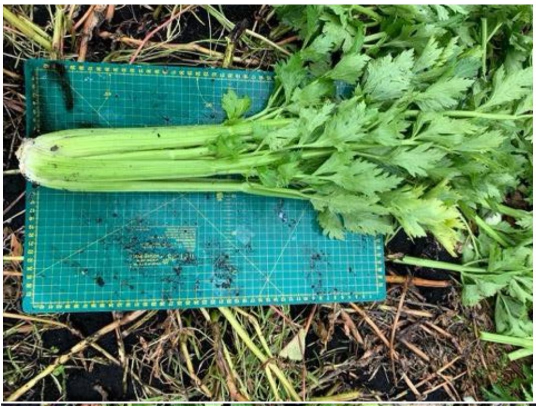
18	EU	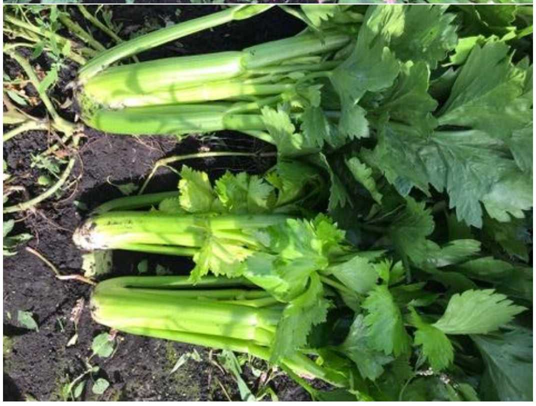	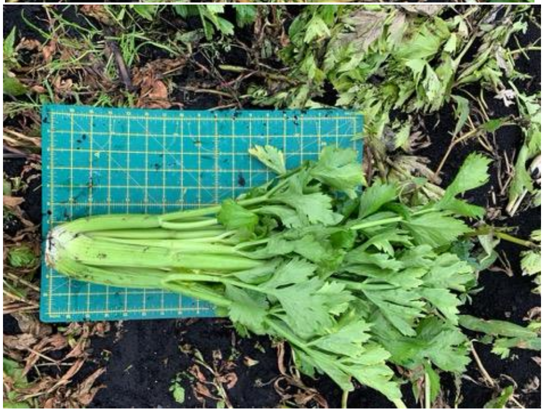
22	USA	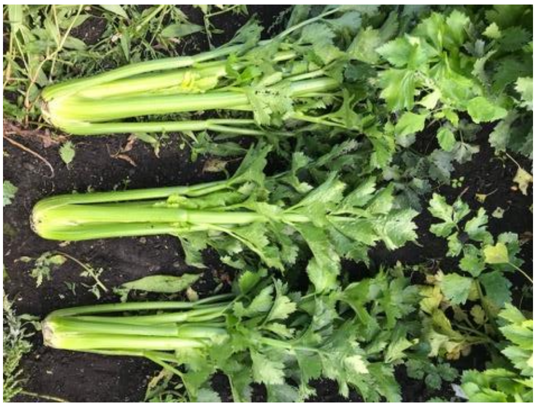	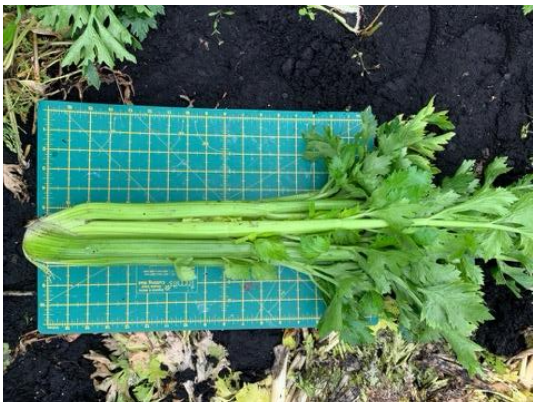
25	EU	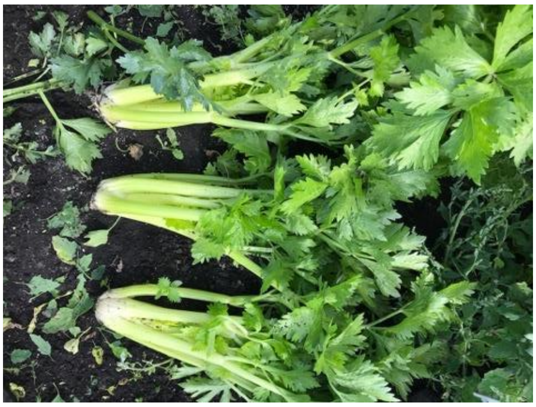	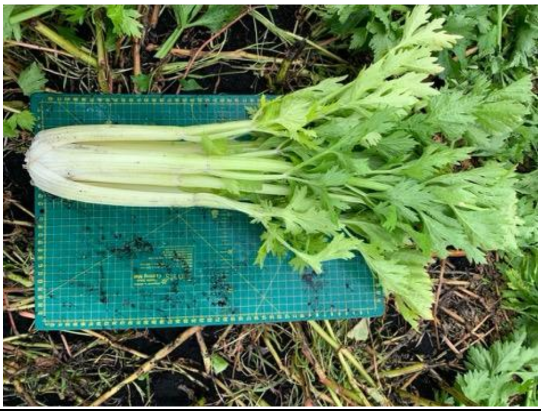
